# Positive digital communication among youth: The development and validation of the digital flourishing scale for adolescents

**DOI:** 10.3389/fdgth.2022.975557

**Published:** 2022-09-01

**Authors:** Jasmina Rosič, Sophie H. Janicke-Bowles, Luca Carbone, Bojana Lobe, Laura Vandenbosch

**Affiliations:** ^1^ School for Mass Communication Research, Faculty of Social Sciences, KU Leuven, Leuven, Belgium; ^2^ School of Communication Research, Chapman University, Orange, California, United States; ^3^ Center for Methodology and Informatics, Faculty of Social Sciences, University of Ljubljana, Ljubljana, Slovenia

**Keywords:** scale development, digital flourishing, digital communication, positive media psychology, well-being, adolescents

## Abstract

Research has extensively studied the negative effects of digital communication on adolescents’ well-being. However, positive digital experiences and behavior in adolescence are still poorly understood. The recently developed Digital Flourishing Scale addresses this gap and focuses on the positive perceptions of a user’s experiences and behaviors in digital communication among adults. In this paper, we developed an adolescent version of this scale. Study 1 demonstrated the internal consistency of the scale and the same factor structure for adolescence as for adulthood: connectedness, civil participation, positive social comparison, authentic self-presentation, and self-control. Study 2 confirmed the identified factor structure with a second sample of adolescents and established measurement invariance across genders. The construct validity of the scale was confirmed by investigating associations with related constructs, including the basic psychological needs from self-determination theory (competence, autonomy, and relatedness), secure attachment to a close friend, Internet aggression, social media-induced inspiration, authenticity of posted positive content, and social media self-control failure. The results indicated that not all adolescents flourish equally online. Differences occurred depending on the adolescents’ gender and socioeconomic status. The paper concludes that the newly developed scale is a valid and reliable measure for assessing adolescents’ perceptions of digital thriving and digital empowerment.

## 1. Introduction

The influence of digital communication technologies, such as social media and smartphones, on the well-being and mental health of adolescents has received considerable research attention. Several instruments have been developed to assess negative perceptions of technology use, including problematic Internet, social media, or mobile use (1–3) and the fear of missing out (4). However, users’ positive perceptions of their technology use are much less explored (5). Several studies have measured the positive effects of digital communication on user well-being using concepts such as self-esteem (6), self-affirmation (7), and, most often, social capital and support (8). Such variables can be considered indicators of perceived positive technology use, yet they do not measure perceived positive digital communication directly or comprehensively.

Thus, while instruments exist that try to identify users who perceive themselves as experiencing harm from digital technology use, we lack a conceptualization and measurement of perceived positive digital experiences and behaviors (i.e., those that thrive in digital communication). This is specifically relevant to adolescents’ technology use. This age group (from 13 to 17 years) is not only using digital communication the most (9) but has also been described in the popular press as the most negatively affected age group, even though new evidence suggests that these negative mental health and well-being effects are highly person-specific (10, 6).

Moreover, not only do effects vary on an individual level, but they are also dependent on the type of use and its measurement (5, 11, 10). Existing scales of perceived communication technology typically measure behaviors or experiences tied to a particular device (e.g., smartphone), application (e.g., social media and email), or feature (e.g., status update and private messenger) rather than experiences or behaviors that are communication centered and thus shared across devices, applications, and features (11, 12). To address these shortcomings in existing measures, the Digital Flourishing Scale (DFS) (13) has recently been proposed, which focuses on perceived digital communication rather than on specific devices or applications. The DFS has been used to study the adult population and has not yet been evaluated in adolescent research. The present research adapted the existing measure to specifically capture digital communication that is perceived to be positive by adolescents. The measure thus introduced a new assessment of the positive perceptions of young media users’ digital communication into the literature. This measure will be henceforth referred to as the Digital Flourishing Scale for Adolescents (DFSA); its validity and reliability were tested in different samples of adolescents. The specific aims of the present research were to (a) identify the factor structure of the newly proposed scale *via* exploratory factor analysis in the first sample of adolescents, (b) evaluate the internal consistencies for the subscale scores, (c) confirm the factor structure with another sample of adolescents *via* confirmatory factor analysis, (d) examine the DFSA for measurement invariance, (e) investigate the construct validity of the DFSA scores with the existing measures, and (f) offer some first results of how the DFSA relates to adolescents’ identity by exploring the relationships of its subscales with demographic variables (i.e., gender, age, education track, paternal and maternal education level).

## 2. Defining digital flourishing

According to Janicke-Bowles et al. (13), digital flourishing is defined as the positive perceptions of an individual’s experiences and behaviors in digital communication. To measure this concept, an instrument was developed for adults that consists of five internally valid digital flourishing dimensions: connectedness (level of perceived connectedness with one’s online network), civil participation (level of considerate digital communication), positive social comparison (level of inspiration from positive online comparisons), authentic self-presentation (level of authentic presentation of the self in digital communication), and self-control (level of control over one’s digital communication).

In general, the DFS captures high digital flourishing as users’ multi-faceted perceptions of the benefits of digital communication. These include individuals feeling closely connected to and supported by their online community, their considerate and reflected interactions with others, knowing how to present themselves consistently and authentically in digital arenas, feeling inspired when compared with others, and being in control of when to start and when to stop interacting online.

The DFS and its background have several characteristics that make this scale a unique instrument in the current literature. First, Janicke-Bowles et al.’ (13) conceptualization of digital flourishing is based on the notion of digital communication, also termed computer-mediated communication, which is defined as “an inclusive umbrella term for multimodal human-to-human social interaction mediated by information and communication technologies (ICT’s)” (11, pp. 2–3, 12). Such digital communication includes interpersonal and masspersonal “active” communication (e.g., instant messaging and posting status updates) as well as more “passive” social attention (e.g., browsing through social media) (14). Research has converged on the preliminary conclusion that the effects of social media on well-being depend strongly on the interactional qualities of its use, specifically whether the use entails active communicative or passive consumptive elements of social interaction (15, 11). Given this centrality of digital communication for well-being—rather than the time spent on the device or other channel-related aspects (16)—the digital flourishing scale focuses on this communication level.

Second, the scale focuses on the positive aspects of mental health, specifically flourishing. Flourishing (17, 18) was first conceptualized in positive psychology and is understood as “feeling well,” which is generally operationalized as subjective well-being (19), and “doing well,” which is also referred to as eudaimonic well-being (20).

Theoretically, it has been argued that flourishing (i.e., subjective and eudaimonic well-being) is determined by the satisfaction of three basic psychological needs: competence, relatedness, and autonomy, as exemplified in self-determination theory (SDT) (21). Furthermore, Gudka et al. (5) referred to these basic psychological needs as important conditions for flourishing within the context of social media. Therefore, the DFS (13) uses SDT as an organizing framework to identify and organize the core facets of digital flourishing. According to SDT, humans are intrinsically motivated to act in the world because doing so will satisfy their basic psychological needs for competence, relatedness, and autonomy, which are essential for short-term hedonic well-being and long-term eudaimonic growth (21). Competence is related to perceiving oneself as effective in manipulating the environment in a way that results in valued outcomes; relatedness involves a sense of connection, care toward others, and feeling cared for by others; and autonomy refers to having a sense of control, volition, or freedom when engaging in an activity (21).

In a first validation study of the DFS scale in adults, the authors (13) found that the five individual subscales of digital flourishing were significantly associated with the satisfaction of the basic psychological needs. This supports the overall notion that the flourishing dimensions are relevant to competence, relatedness, autonomy, and, ultimately, well-being.

## 3. Digital flourishing in adolescence

Adolescence is a life stage with several psychosocial and cognitive developmental changes that clearly distinguish it from other life stages (22). Psychosocial changes include continuous emotional separation from parents and the increased importance of socializing with peers and other socialization agents (23). Cognitive changes include improved cognitive self-regulation, increased emotion regulation, and impulse control (24).

These psychosocial and cognitive changes affect the satisfaction of the basic psychological needs (i.e., relatedness, competence, and autonomy), as defined by SDT (21). First, the need for relatedness leads adolescents to bond more strongly with peers (rather than parents) (23). Second, the need to feel competent leads them to take up more challenging cognitive tasks in line with their growing cognitive skills (24). Third, psychosocial and cognitive changes also lead adolescents to satisfy their need for autonomy in different ways than in preadolescence (e.g., striving toward more advanced tasks that satisfy the feeling of independence) (25).

The changed ways in which adolescents meet their basic psychological needs are also expressed in adolescents’ differential uses of digital communication (i.e., connecting with friends, self-presentation, social comparison, civil participation, and controlled use). These uses provide unique opportunities to satisfy adolescents’ basic psychological needs and flourish online (26). In adolescence, digital communication is, for instance, a more central way of communicating and connecting with peers and others than in other life stages (27).

More precisely, psychosocial changes prompt adolescents to engage in digital communication, as digital communication offers a multitude of opportunities to build stronger connectedness with online and offline peer communities (28). Research has demonstrated that adolescents seize online opportunities and engage in digital communication to strengthen their bonds with existing friends and build new friendships online (29). Behaviors such as posting images on public social media (30), privately exchanging messages and photos, or sharing everyday information and small signs of affection throughout the day often extend adolescents’ offline communication and strengthen their relationships with their peers (31).

In addition to enhancing connectedness, digital communication allows adolescents to self-present and become more autonomous individuals. In this view, adolescents have stressed the importance of remaining authentic in their self-presentation when sharing information about themselves (32, 33). Such authentic self-presentation is sometimes challenged, given that positivity norms and platform features, such as filters, invite adolescents to present the best (but not necessarily true) version of themselves (34, 35). Yet, presenting one’s true self online is known to be beneficial for adolescents’ psychological development, leading to increased self-esteem (36), which is, in turn, important for well-being.

Digital communication also invites adolescents to participate in social comparison processes that inform them about how competent they are in relation to their peers. Noon and Meier (37) argued that most studies on digital social comparison particularly focused on whether upward comparisons evoked jealousy, malicious envy, anxiety, and increased depressive symptoms among adolescents. Recent research has also indicated that positive social comparisons (i.e., comparisons to authentic and similar others) occur in adolescence and may evoke feelings of motivation, inspiration, enjoyment, and benign envy; this motivates adolescents to self-improve (37, 33).

Similarly, research has focused on uncivil online participation in adolescence, mostly in the context of cyberbullying, but has neglected the advantages that come with civil online participation (38). Civil participation may be especially relevant for adolescents, as psychosocial changes toward stronger bonding with peers encourage them to engage more actively in online discussions; thus, civil participation can address their increased need for relatedness (39). Cognitive changes in emotion regulation and impulse control make it possible for these online discussions to be conducted in a respectful, polite, mindful, responsible, and civil manner (40). Some research has shown that adolescents are mindful of civil participation in online communities (41).

Lastly, cognitive changes contribute more to increased self-control over digital communication in adolescence than in younger ages (children aged 11 years and younger) (42). The digital flourishing dimension of self-control is particularly relevant to contemporary adolescents who are constantly connected to their digital devices. They are more challenged to control when and how often they connect, and thus how autonomous they feel in their digital connection. Having such self-control has been related to positive outcomes, including positive well-being (15) and increased cognitive performance (43).

In sum, research has focused on the relationship between digital communication in adolescents and digital flourishing. Yet, digital flourishing has never been systematically examined as a comprehensive concept among adolescents. Most studies have also focused on the negative effects of digital behaviors on adolescents, leaving positive aspects often unconsidered.

## 4. The current study

To date, we lack an instrument that captures digital flourishing in adolescence. Therefore, the present research adapted the existing Digital Flourishing Scale (13) to adolescents’ (DFSA) digital communication experiences and behaviors. Two cross-sectional studies were organized among adolescents to explore and confirm the factor structure of the new instrument, to test for measurement invariance, and to establish the construct validity of the scale (as a whole and for respective subscales). An overview of the included validation concepts is given below.

### 4.1. The satisfaction of basic psychological needs

The concept of digital flourishing is grounded in SDT (21). According to SDT, adolescents with satisfied basic psychological needs of autonomy, competence, and relatedness are more likely to act prosocially and have higher well-being (25). The authors of the DFS (13) used the psychological need satisfaction scale (21) in their validation study and found that all five flourishing dimensions were significantly associated with the satisfaction of basic psychological needs. As such, we tested whether positive correlations could be found between the three satisfied basic psychological needs and the newly developed DFSA.

### 4.2. Technology interference

Technoference refers to interruptions to offline social interactions due to technology use (44). The concept is based on the premise that during an offline social interaction, one interaction partner uses (or starts using) technology, keeps this content to themself, and thus interrupts the conversation (45). Experimental research demonstrated that technoference among strangers and close friends (emerging adult dyads) was related to decreased feelings of closeness, lower interpersonal connectedness, reduced quality of interactions, and decreased friendship intimacy (46, 47). Accordingly, we expected that higher rates of technoference in adolescents would be negatively correlated with the DFSA dimension(s) of feeling connected to close and distant others online.

### 4.3. Posting positive social media content

Adolescents are often exposed to positivity bias on social media. Consequently, some adolescents feel pressured to post socially desirable (positive) portrayals of their lifestyles online (34, 35). Schreurs and Vandenbosch (34) identified three main types of positively biased content posted by adolescents on social media: attractive appearance, happy and interesting social life, and (professional) achievements. These contents are not always authentic, especially when adolescents frequently post positively biased posts (34). Accordingly, we assumed that higher rates of posting positively biased social media content would be negatively related to the DFSA dimension(s) of being authentic in one’s self-presentation online.

### 4.4. Social media-induced inspiration

Prevalent positive presentations of users on social media prompt adolescents to compare themselves with the achievements of others posted online (i.e., upward comparisons). These upward comparisons can evoke positive feelings of inspiration (48). One study showed that the more adolescents compared themselves online, the more inspiration they experienced. In addition, comparisons were more inspiring when adolescents compared themselves to similar others who authentically presented themselves online (i.e., positive social comparison) (37). Therefore, we expected that a positive relationship would emerge between social media-induced feelings of inspiration and the DFSA dimension(s) related to positive social comparisons in digital communication.

### 4.5. Aggressive digital communication

Internet aggression refers to intentional online aggressive behavior toward others. It comprises rude, embarrassing, threatening, or harassing comments, unwanted sexual comments, and exclusion (e.g., blocking someone’s messages) (49). This concept stands in strong contrast to online civil participation. The latter concept refers to responsible, mindful, open, and polite digital communication, which includes discussions between people with different points of view (13). Internet aggression is the opposite of civil participation; therefore, we expected a negative correlation between Internet aggression and the DFSA dimension(s) of civil participation.

### 4.6. Social media self-control failure

Social media users sometimes fail to control their temptation to use social media when it conflicts with other goals and obligations. Digital interruptions (e.g., notifications) typically conflict with pursuing other goals (e.g., professional or educational achievements), making efficient use of time, and performing important tasks (50). It can be assumed that adolescents who fail to self-control social media use are also challenged by controlling other digital communication (e.g., texting) (51). Hence, we expected that social media self-control failure would negatively correlate with the DFSA dimension(s) of self-control.

## 5. General method

We followed the methodological standards of the scale development literature to develop the DFSA (52). First, the DFS was adapted to apply to adolescents. The DFS begins with an introduction explaining the term ‘digital communication’. Within the DFSA, the original introduction was adapted by using language suitable for adolescents and adding specific examples of digital communication platforms (e.g., having an interaction on social media was illustrated with the example of “posting, commenting or liking posts on SnapChat, Instagram, TikTok or YouTube”). Moreover, as the methodological literature recommends using 5-point scales and not 7-point scales with adolescents (53), the original response format of the DFS (i.e., a 7-point Likert scale) was adapted to a scale ranging from 1 (not at all true of me) to 5 (very true of me) with an option “not applicable to me”. The original Likert scale to measure agreement was discouraged to be used by the authors of DFS because it assesses general (dis)agreement with the statements (13) rather than how true each behavior or experience is for the person. Therefore, we used “true-of-me” answer options (53). Additionally, authors have recommended using more concrete time periods as unspecified timeframes cause confusion more easily (13). As such, adolescents were asked to assess each item as it had applied to them in the last month.

In line with the DFS, the newly proposed DFSA also contained a 25-item scale with 5 expected subscales including 5 items each. These subscales were intended to measure the dimensions of connectedness, civil participation, positive social comparison, authentic self-presentation, and self-control. Each subscale in the newly developed DFSA started with an introductory sentence in the child-friendly language (e.g., the civil participation subscale began with “The following statements are about how you express your opinion online. When assessing the statements think about the past month.”). The existing items were adapted to be suitable for contemporary adolescents in terms of the language used and their developmental level. For example, we used simpler terms and included several specific examples in items. For instance, “When I interact with others about politics online, I know how to have a civil discussion.” was changed to “When I talk to others online about politics (e.g., about the government, the President, elections), I know how to do it politely.”

After these changes, the newly developed DFSA was prepared to be tested in two samples of adolescents. Given the researchers’ accessibility to Slovenian adolescents, the English version of the scale was translated to Slovenian by two English-Slovenian bilinguals using a forward-and-back translation procedure (54). Next, two of the authors that are native Slovenian speakers reviewed the forward-and-back translations. The discrepancies between the Slovenian and English versions of the novel scale were resolved. Semantic, idiomatic, experiential, and conceptual equivalence between these two versions was achieved and consensus on the finalized Slovenian version was reached.

Following this translation, the readability and clarity of the scale were tested in a cognitive interview using a hybrid model (55) with one 15 years old female adolescent. Based on the feedback of this interview, we adapted the instructions (e.g., by adding Viber and TikTok as examples) and some items to be more suitable for early, middle, and late adolescents. For instance, a concrete example of acquaintances was added in the item: “I could turn to people who I connect with online (e.g., acquaintances) if I needed advice on a problem.” The interviewed adolescent also confirmed she found the use of the “true-of-me scale” clear and supported the idea of using a time frame for assessing the items. An online Supplementary Appendix A in OSF presents the adaptation of DFS to DFSA.

In the next step, two cross-sectional surveys were organized. Study 1 examined which exploratory factor structure emerged in the adolescent population and determined the internal consistency of the newly developed DFSA. The purpose of Study 2 was to confirm the factor structure of the DFSA with a different sample of adolescents and examine measurement invariance across gender. Across the two studies, we also examined the associations between the newly identified digital flourishing dimensions with the constructs outlined above to explore the construct validity of the DFSA.

Supplementary Appendixes B and E in OSF display the initial and final items in English and Slovenian used in both studies. Data and other supplementary materials of both studies are also available online (https://osf.io/9wuyb/?view_only = 9e64aa7358ed40a0823a8cb75c49c3ae). Both studies were approved by the ethical commission of KU Leuven, Belgium, and University of Ljubljana, Slovenia.

## 6. Study 1

### 6.1. Method

#### 6.1.1. Sample and procedure

For the first cross-sectional online survey a combined purposive sample of Slovenian adolescents (aged between 16 and 19) was recruited in June-August 2021. Four different recruitment strategies were applied. First, several secondary schools from different regions and educational tracks were contacted and five agreed to participate. Schools presented the aims of the study and provided adolescents with information brochures and parental consent forms (*via* an online link or paper versions). For adolescents younger than 16, parents completed an active consent form, for adolescents aged 16 and older, passive parental consent was requested. Participants gave active consent to participate before the start of the survey. A total of 144 adolescents were recruited *via* the schools. The majority of participants [*N* = 131] completed the survey in class, and 13 completed it at home. Second, the sample was further extended by recruiting adolescents *via* 20 youth organizations (youth centers and scout movement organizations; the latter organizations are youth movements organizing practical outdoor leisure activities for youth aged 6–30 years old). These organizations invited their adolescent members aged 16–19 to participate in the study by providing them with information brochures, active consent sheets, and informing them of the passive parental consent procedure. Third, Facebook advertising was used by targeting the parents of adolescents aged 16–20 years and asking them, after giving passive consent, to invite their children to participate in the study. Again, adolescents were informed *via* information brochures and gave active consent. A total of 31 adolescents were recruited *via* youth organizations and Facebook advertising. Lastly, the first author also invited adolescents from her personal network [*N* = 7]. Adolescents were informed *via* information brochures and active/passive parental consent was requested depending on the age of the adolescent. They also gave active consent to participate before entering the survey. Participants were rewarded with a 5-euro voucher. Adolescents outside the age range of 11–20 years, without parental consent, and non-smartphone users could not participate. A total of 182 participants took part in the survey. Participants who had missing data on all items of the new scale were deleted [*N =* 35]. The final sample consisted of 147 participants aged 12–20 (*M_age_* = 17.90, *SD* = 1.24, 59.18% girls). Based on the Slovenian secondary school system division, 72.80% followed the general education in which they were being prepared for college education, 19.73% followed the professional-technical education in which they were being taught primarily technical and professional skills, and 3.40% followed the vocational education leading to professions (e.g., merchant, carpenter); 4.08% were in elementary schooling or in higher education. The majority (67.35%) described their ethnicity as only central European (Slovenian). Within the sample, 49.66% of participants’ mothers had a university degree and 46.94% of fathers had secondary education.

#### 6.1.2. Measures

Measures were translated from English to Slovenian following a forward-and-back translation procedure. Half of the scales used to estimate construct validity were displayed to one subsample and the other half to the other subsample to avoid survey fatigue. Some of the validation scales (and the DFSA) offered an option “not applicable to me”. Respondents who answered this option were coded as having a missing value. Because of the varying number of respondents, the exact number of respondents for each variable is reported in Table 2. Reliability of scales was interpreted as acceptable if Cronbach’s *α* ≥ 0.7, as good if *α* ≥ 0.8, and as excellent if *α* ≥ 0.9 (56). The online Supplementary Appendix B in OSF displays full items of the used scales.

##### 6.1.2.1. Demographic variables

Adolescents reported their age (2021–birth year), gender (1 = boy, 2 = girl, 3 = other, 4 = prefer not to say; categories 3 and 4 were coded as a missing value), educational track (1 = vocational, 2 = professional-technical, 3 = general education), a parental education level (measured separately for mother or female guardian and father or male guardian with 5 categories; educational categories were coded as < 4 = secondary education and lower, labeled low education, and >= 4 = post-secondary education, labeled high education), and ethnicity [with the categories allowing to choose multiple options (e.g., Central European, West European, South-East European)].

##### 6.1.2.2. Digital flourishing in adolescence

The 25-item Slovenian DFSA ranging from 1 (not at all true of me) to 5 (very true of me) with an option “not applicable to me” was used.

##### 6.1.2.3. The satisfaction of basic psychological needs

To measure self-determination in adolescence, we used the Satisfaction of Basic Psychological Needs in Adolescents questionnaire (25) with a 5-point scale ranging from 1 (not at all true of me) to 5 (very true of me). The scale consists of 12 items (e.g., “I feel good at doing many things.”) with three subscales. The subscales yielded acceptable to good internal consistencies: Autonomy (*α* = 0.76, 4 items, *M *= 3.9, *SD* = 0.70), Relatedness (*α* = 0.83, 4 items, *M* = 3.9, *SD* = 0.65), Competence (*α* = 0.74, 4 items, *M *= 3.9, *SD* = 0.64).

##### 6.1.2.4. Technology interference

We used the modified version of the Technoference Scale (57). The original word “parents” was replaced with “friends” to align the content with our scale. Respondents rated three statements (e.g., “I ignore my friends when I am on my tablet/cell phone”) on a 5-point scale ranging from 1 (not at all true of me) to 5 (very true of me), with higher scores indicating higher levels of technoference. The scale displayed average reliability considering it only included 3 items (*α* = 0.58, 3 items, M = 2.1, SD = 0.68).

##### 6.1.2.5. Posting positive social media content

We used the Posting Positive Social Media Content short form scale (34). Answers ranged from 1 (never) to 5 (very often), with the option “not applicable to me”. The scale consisted of 8 items (e.g., “How often do you post on most public applications, such as social media, posts in which you look beautiful.”) and three subscales: Attractive Appearance (correlation between 2 items: *r* = 0.69, *p* < 0.001, *M* = 2.1, *SD* = 1.0), Happy (Social) Life (*α* = 0.90, 5 items, *M* = 2.5, *SD* = 1.0), (Professional) Achievements (1 item, *M* = 2.0, *SD* = 0.97).

##### 6.1.2.6. Social media-induced inspiration

We used the two items of the Social Media-Induced Inspiration Scale (48): “When I use social media I am inspired by the posts of other users to do something [new].” and “When I use social media I experience inspiration.” The word “Instagram” was replaced with “social media”. Answers ranged from 1 (strongly disagree) to 5 (strongly agree) with the option “not applicable to me”. A strong correlation between the two items was found (*r* = 0.70, *p* < 0.001, *M* = 3.0, *SD* = 0.98).

##### 6.1.2.7. Aggressive digital communication

We used the 4-item Internet Aggression Scale (49) (e.g., “I used the Internet to play a joke or annoy someone I was mad at.”). Answers ranged from 1 (never) to 4 (5 or more times) with the option “not applicable to me”. This scale displayed acceptable reliability (*α *= 0.77, 4 items, *M* = 1.2, *SD* = 0.47).

##### 6.1.2.8. Social media self-control failure

We used the three items of the Brief Measure of Social Media Self Control Failure (50) (e.g., “How often in the past month did you give in to a desire to use social media even though your social media use at that particular moment made you use your time less efficiently?”). Answers ranged from 1 (never) to 5 [very often (10 or more times)] with an option “not applicable to me”. The scale displayed good reliability (*α* = 0.85, 3 items, *M* = 3.1, *SD* = 0.97).

#### 6.1.3. Analytical strategy

We followed the analytical strategy applied in prior scale development literature (e.g., 52, 34, 65) and first explored the factor structure of the DFSA. First, a principal component analysis (PCA) was conducted on the 25 items in R (version 4.0.4) to evaluate the number of components. The number of components to extract was determined on the basis of the Kaiser’s criterion (eigenvalue > 1), examining the scree plot, percentage of variance accounted for per component [total cumulative variance explained (50%)], and parallel analysis (52). Second, we run an exploratory factor analysis (EFA) based on the number of factors selected through PCA and looked at the loadings of the items on each factor. Given that a correlation between sub-factors was expected, an oblique rotation (Promax method) was used. Items were removed due to either (a) item-factor loading below 0.5 on a primary factor, (b) factor loadings on multiple factors (above 0.3) or on a theoretically wrong factor, or (c) low communalities (below 0.4) (58). After omitting items, the process of running the EFA was repeated until item loadings were satisfactory, as factor loadings and structure can change after removing items.

After exploring the factor structure of the DFSA, internal consistency of the subscales was assessed by calculating Cronbach’s alpha values for each identified (sub)factor with three or more items.

Finally, bivariate Pearson correlations were computed between the newly developed (sub)factors and selected construct validity variables to investigate construct validity as well as between the DFSA (sub)factors and demographic variables (i.e., gender, education track, paternal and maternal education level). Correlations were considered weak if values of *r* 0.10 ≤ 0.30, moderate *r* 0.30 ≤ 0.50, and strong *r* ≥ 0.50 (59).

### 6.2. Results

#### 6.2.1. EFA

The size of the Kaiser-Meyer-Olkin measure of sampling adequacy (KMO = 0.75) and Barlett’s test of sphericity, *χ^2^*(300) = 1757.086, *p* < 0.001, suggested that the data was factorable.

In line with the original DFS, the initial factor structure results of the EFA also suggested a five-factor structure explaining 56.3% of variance. Also, a sudden drop in the scree-plot was seen after five factors were reached, and a parallel analysis supported this factor structure. The items that clustered on the same factors showed that factor 1 represented authentic self-presentation (eigenvalue = 6.60, 13.5% of the variance, 5 items, *M* = 3.4, *SD* = 0.87), factor 2 represented positive social comparison (eigenvalue = 3.40, 11.4% of additional variance, 5 items, *M *= 3.2, *SD* = 0.83), factor 3 represented civil participation (eigenvalue = 2.85, 11,1% of additional variance, 5 items, *M* = 4.0, *SD* = 0.70), factor 4 represented connectedness (eigenvalue = 2.05, 10.9% of additional variance, 6 items, *M* = 3.2, *SD* = 0.75), and factor 5 represented self-control (eigenvalue = 1.95, 9.4% of additional variance, 4 items, *M* = 3.7, *SD* = 0.67).

Four items were deleted after the initial inspection of factor scores (i.e., three items due to low factor loadings and one item due to conceptual incoherence with its primary factor: an item on self-control loaded on the connectedness factor) (see Supplementary Appendix C in OSF for a table with an initial examination of factor structure).

An EFA after deleting these items confirmed a five-factor model with improved results, explaining 60.8% of the variance with a total of 21 items and the same factors (*M* = 3.4, *SD* = 0.49). Table 1 shows the final items and their factor loadings, communalities, eigenvalues, explained variance, and descriptive statistics. A five-dimensional factor structure with five separate, yet related latent factors was thus confirmed.

**Table 1:** Number of respondents, communalities, rotated factor loadings, reliability and descriptive statistics of the final DFSA items (study 1)..

	N	Communalities	Factor 1	Factor 2	Factor 3	Factor 4	Factor 5
			Authentic self-presentation	Civil participation	Positive social comparison	Connectedness	Self-control
1. I show my true self online.	131	0.524	0.789				
2. When communicating online, I feel comfortable presenting the person I am.	131	0.599	0.771				
3. What I post online reflects who I really am.	126	0.493	0.781				
4. I feel comfortable presenting who I truly am online, in the same way I do offline.	133	0.665	0.794				
5. I allow people who I connect with online to see who I really am.	130	0.533	0.799				
6. When I talk to others online, I know how to share my point of view without offending them.	134	0.332		0.614			
7. When I communicate online, I am careful to adapt my comments and behaviors to be appropriate for whoever will read them (e.g., my friends, my teacher, my parents, younger children).	138	0.530		0.847			
8. When I talk to others online about something important to me, I know how to stand for it in a polite manner.	135	0.378		0.602			
9. When I talk to others online about politics (e.g., about the government, the President, elections), I know how to do it politely.	100	0.604		0.818			
10. When something that others say or do online makes me feel angry, I am able to respond in a calm way.	129	0.482		0.711			
11. Seeing others’ achievements online inspires me to do better.	138	0.565			0.847		
12. Seeing how others present themselves online motivates me to make changes in my own life.	137	0.745			0.882		
13. Comparing myself to others online motivates me to accomplish the things I want in life.	138	0.616			0.684		
14. I compare my life to those people online (e.g., peers, influencers) who are going to push me to be better.	134	0.439			0.649		
15. I feel part of a group when I communicate with others online.	139	0.741				0.880	
16. I find my online communication (e.g., chatting with peers, playing online games with others) very important.	141	0.580				0.844	
17. I feel closely connected to the groups I connect with online.	139	0.431				0.682	
18. I feel in control of when to start and when to stop spending time on online communication.	134	0.409					0.626
19. For the most part, I feel in control of how much time I spend communicating with others online (e.g., chatting with friends, posting on Instagram, playing online games with others).	135	0.414					0.756
20. When I browse through online content, I feel in control of how I spend my time.	138	0.278					0.632
21. I am able to disconnect from my online communication when I need a break.	136	0.422					0.688
Eigenvalues			5.813	3.281	2.752	1.687	1.513
% of variance			15.6	13.3	12.1	10.3	9.5
M (SD)			3.4 (0.87)	4 (0.70)	3.2 (0.86)	3.1 (0.94)	3.7 (0.67)
Cronbach’s *α*			0.86	0.81	0.85	0.80	0.70

Factor correlations ranged between *r* = 0.21 to 0.54, indicating that the subscales represent distinct dimensions of digital flourishing (see Supplementary Appendix D in OSF for correlations table).

#### 6.2.2. Internal Consistency

Cronbach’s *α*’s for the final factors were 0.86 for authentic self-presentation, 0.85 for positive social comparison, 0.81 for civil participation, 0.80 for connectedness, and 0.70 for self-control. These findings support the internal consistency of the DFSA’s scores.

#### 6.2.3. Construct Validity

The flourishing dimensions correlated significantly with the bulk of the validation constructs included (see Table 2; no correlations are reported in text; all can be found in Table 2). First, all subscales were moderately but significantly associated with at least one if not more basic SDT needs (i.e., competence, relatedness, autonomy). The connectedness subscale correlated with all three needs, most significantly with relatedness. The civil participation subscale and the self-control subscale were the most significantly associated with autonomy. Finally, the positive social comparison and authentic self-presentation subscales most significantly correlated with competence.

**Table 2:** DFSA construct validity and correlations with demographic variables (study 1).

	Connectedness		Civil participation		Positive social comparison		Authentic self-presentation		Self-control	
	N	r	N	r	N	r	N	r	N	r
Autonomy	68	0.25*	54	0.36*	59	0.13	61	0.33**	66	0.27*
Competence	68	0.24*	54	0.26	59	0.31*	61	0.40***	66	0.23
Relatedness	68	0.29*	54	0.30*	59	0.28*	61	0.26*	66	0.19
Technoference	63	−0.08								
Internet aggression			51	−0.33*						
Social media-induced inspiration					60	0.66***				
PPSMC: happy and interesting (social) life							51	0.17		
PPSMC: attractive appearance;							49	0.16		
PPSMC: (professional) achievements							59	−0.19		
Social media self-control failure									63	−0.41***
Gender (girls is ref.category)	132	−0.08	94	0.32**	129	−.07	118	0.03	131	−0.04
Education track (vocational is ref.category)	129	0.05	95	0.30**	126	.04	114	−0.17	126	−0.17
Education track (vocational)	5	10.6 (2.51)^a^	5	16.8 (4.60)^a^	5	12.20 (2.77)^a^	4	21.5 (2.65)^a^	4	17.5 (1.91)^a^
Education track (technical)	28	8.46 (2.53)^a^	20	18.35 (3.38)^a^	26	12.46 (3.17)^a^	27	17.3 (3.82)^a^	25	14.8 (2.61)^a^
Education track (general)	96	9.44 (2.91)^a^	70	20.34 (3.39)^a^	95	12.74 (3.72)^a^	83	16.64 (4.63)^a^	97	14.4 (2.63)^a^
Education father (high is ref.category)	121	0.03	87	0.02	115	−0.00	108	−0.18	120	−0.04
Education father (low)	72	−0.06	51	0.17	68	0.08	65	−0.04	74	0.04
Education father (high)	49	−0.11	36	0.28	47	0.01	43	−0.04	46	−0.04
Education mother (high is ref.category)	123	0.08	89	−0.01	119	−0.19	110	−0.03	122	−0.04
Education mother (low)	44	0.07	29	−0.26	43	−0.19	42	−0.10	44	−0.09
Education mother (high)	79	−0.03	60	0.06	76	−0.17	68	0.01	78	0.11

Note. *p < 0.05, **p < 0.01, ***p < 0.001.

PPSMC: Posting positive social media content.

^a^ Means instead of correlation.

Low education: secondary and lower; high education: post-secondary.

As expected, the civil participation scale was moderately negatively correlated with the Internet Aggression Scale. The subscale of positive social comparison correlated strongly with the Social Media-Induced Inspiration items. A moderate negative association was found between the subscale on self-control and the Brief Measure of Social Media Self-Control Failure.

However, the subscale on connectedness did not correlate with the proposed Technoference Scale. We also did not find correlations between the subscale of authentic self-presentation with the proposed subscales of the Posting Positive Social Media Content Scale.

The relationships between the DFSA subscales and the demographic variables were also explored. There was a significant moderate correlation between the civil participation subscale and gender, indicating that girls demonstrated higher scores on civil participation than boys (see Table 2). The civil participation subscale was also significantly moderately correlated with adolescents’ secondary education track. Adolescents following general education demonstrated higher scores than adolescents following professional-technical and vocational education.

### 6.3. Brief discussion of study 1

The results of Study 1 preliminary confirmed the five distinct dimensions of DFSA and indicated good reliability of all the identified subscales.

As for validity, the five flourishing subscales were significantly associated with the satisfaction of basic psychological needs. The subscales of civil participation, positive social comparison, and self-control were also validated by showing correlations with scales of Internet aggression, social media-induced inspiration, and social media self-control failure, respectively.

Yet, authentic self-presentation was not related to posting positive content on social media. The reason for this result might be that the measure of positive posts does not allow to distinguish whether the posts with positive content are (in)authentic (34). The measure asks, for instance, how often adolescents post content on social media in which they look beautiful, or do something fun. For some adolescents, this type of self-presentation is a genuine reflection of their lives. For other users, these posts might be strategically selected to present the best and therefore also a biased and more unauthentic version of the self. The measure of positive content on social media thus does not allow making a claim on the authenticity/inauthenticity of the adolescents’ online self-presentation. Therefore, in Study 2 an alternative measure that could distinguish the authenticity of posted positive content was used. Finally, we did not find the expected negative relationship between technoference with friends and online connectedness. The possible explanation for this non-significant finding might lie in the changed norms of technology use in offline conversations. Using devices for digital communication during offline conversations has become more socially accepted (60) and adolescents indicate to perceive technology use during face-to-face conversations as a complementary extension of the ongoing conversation (61). To validate the connectedness subscale in Study 2, we, therefore, searched for another validation concept in the attachment literature. Individuals with a secure attachment are reasoned to be more likely to form close social connections with others online (62) and to use digital communication more often to satisfy their need for relatedness (63). We thus expected that adolescents with a secure attachment to their peers would experience higher levels of connectedness with people online.

## 7. Study 2

### 7.1. Method

#### 7.1.1. Sample and Procedure

The preregistered Study 2 was conducted in September-November 2021 as the first wave of a larger, three-wave longitudinal study of the ‘MIMIc Project’, focusing on media and well-being.^1^ A quota sample of 1,168 adolescents was recruited through 27 elementary and secondary schools in Slovenia, considering a stratified distribution of participants’ age, gender, educational track, and region of residence. The schools were selected from an overview of all existing schools provided by the government and were initially contacted with the request to participate in the study. Participating schools next presented the aims of the study to participants, provided parental consent forms to their pupils, and helped with the dissemination of the online survey link and active consent sheet. The majority of the participants [*N* = 727] completed the survey in class, and 441 participants completed it at home. Participants and their parents were informed of the confidentiality and anonymity of the data collection. Active (< 16 years) or passive parental consent (≥ 16 years) was collected prior to the data collection, and active consent of the adolescents themselves at the moment of the data collection. Participants were rewarded with a 10-euro voucher. Adolescents aged outside the range of 11–20 years and without parental consent could not participate. Respondents who failed or had missing data on the attention check [*N =* 101] or had missing data on all items of the DFSA [*N =* 25] were excluded. The attention check question is available in Supplementary Appendix E in OSF. The final sample consisted of 1,046 adolescents (11–18 years, M_age _= 15.28, SD = 1.79, 49.1% boys). Based on the Slovenian secondary school system division, 18.83% followed general education, 28.20% followed professional-technical education, and 20.27% followed vocational education; 32.7% were in elementary schooling. The majority (75.62%) described their ethnicity as only central European (Slovenian). Within the sample, 39.20% of participants’ mothers had a university degree and 42.73% of fathers had secondary education.

#### 7.1.2. Measures

The measures were translated from English to Slovenian following a forward-and-back translation procedure. The online Supplementary Appendix E in OSF displays full items of the used scales.

##### 7.1.2.1. Demographic variables

Adolescents’ age, gender, educational track, ethnicity, and parental education level, were measured using the same scales as Study 1.

##### 7.1.2.2. Digital flourishing in adolescence

The 21-item Slovenian DFSA ranging from 1 (not at all true of me) to 5 (very true of me) with an option “I don’t know/Not applicable to me” was used (*α* = 0.83, *M* = 3.5, *SD* = 0.48). All subscales yielded acceptable to good internal consistency: Connectedness and Civil Participation (*α* = 0.75, *M*_connectedness_ = 3.2, *SD*_connectedness_ = 0.84, *M*_civil _= 3.7, *SD*_civil _= 0.71), Self-Control (*α* = 0.80, *M* = 3.6, *SD* = 0.74), Positive Social Comparison (*α* = 0.81, *M* = 3.3, *SD* = 0.82), Authentic Self-Presentation (*α* = 0.87, *M* = 3.4, *SD* = 0.83).

##### 7.1.2.3. Secure attachment with a close friend

We used the Secure Attachment Style subscale of the short form of the Adolescent Friendship Attachment Scale (e.g., “I can trust my friend”) (64). Participants thought of the peer that they feel closest to and rated 5 items on a 5-point Likert scale ranging from 1 (strongly disagree) to 5 (strongly agree). The scale displayed good reliability (*α* = 0.88, 5 items, *M* = 3.5, *SD* = 0.67).

##### 7.1.2.4. Authenticity of posted positive content

Drawing on the virtual self subscale of Psycho-Social Aspects of Facebook Use (65) one item was developed to evaluate how often respondents had the impression that their posts and stories with positive content on social media showed who they really are. A 5-point Likert scale ranging from 1 (never) to 5 (very often) was used (*M =* 3.0, *SD =* 1.27).

#### 7.1.3. Analytical Strategy

A confirmatory factor analysis (CFA) was conducted in R (version 4.0.4) using the “lavaan” package to confirm the five-factor measurement model of the 21-item DFSA. A Maximum Likelihood estimation was used for exact fit and four goodness-of-fit-indices (i.e., RMSEA, CFI, TLI, and SRMR) for approximate fit (66). Generally, CFI and TLI values between 0.90 and 0.95 and RMSEA values between 0.05 and 0.08 indicate an acceptable model fit, and CFI and TLI values above 0.95 and RMSEA values below 0.05 indicate good model fit (67). SRMR values below 0.08 indicate an acceptable model fit (66) and SRMR values below 0.05 indicate a good model fit (68). The latent sub-factors were allowed to correlate with each other.

Next, measurement invariance across gender (i.e., boys vs. girls) was examined by conducting a multigroup structural equation modeling using Maximum Likelihood as an estimation method. If measurement invariance can be demonstrated, then girls and boys interpret the items and the underlying latent factor, in the same way. First, the five-factor-solution model was tested in each group separately to see if the model solution fitted well the data for each group separately. Second, configural, metric, and scalar invariance were considered to test differences between boys and girls. Configural invariance indicates the same factor structure, metric invariance indicates the same factor structure and loadings, scalar invariance indicates the same factor structure and loadings, and the same item intercepts. The *χ*2 and the AIC of the previous model were constantly compared to those from the following model. Non-significant (*p* > 0.05) *χ*2-differences confirm the invariance of the model.

Internal consistency, construct validity, and correlations with demographic variables were investigated by following the same procedure as in Study 1.

### 7.2. Results

#### 7.2.1. CFA

The CFA indicated a good model fit for the five separate but correlated factor structure [*χ*^2^ (179) = 420.661, *p* < 0.001; RMSEA = 0.045, CFI = 0.951, TLI = 0.942, SRMR = 0.048]. The correlation between the latent variables ranged from *r* = 0.10 to 0.30 (see Supplementary Appendix F in OSF for correlations table).

#### 7.2.2. Measurement Invariance

The five-factor solution showed an acceptable model fit when being tested separately among boys [*χ*^2^ (179) = 382.682, *p* < 0.001; RMSEA = 0.060, CFI = 0.913, TLI = 0.898, SRMR = 0.064] and girls [*χ*^2^ (179) = 277.266, *p* < 0.001; RMSEA = 0.041, CFI = 0.960, TLI = 0.953, SRMR = 0.052]. Next, the configural invariance between boys and girls was confirmed [*χ*^2^ (358) = 659.948, *p* < 0.001; RMSEA = 0.051, CFI = 0.937, TLI = 0.926, SRMR = 0.055]. Then we achieved an acceptable model fit for metric invariance [*χ*^2^ (374) = 684.419, *p* < 0.001; RMSEA = 0.051, CFI = 0.935, TLI = 0.927, SRMR = 0.057]. The *χ*^2^-difference test between the configural model and the metric invariance model was not significant [*χ*^2^ (16) = 24.471, *p* = 0.078]. We therefore tested for scalar invariance which indicated an acceptable model fit [*χ*^2^ (390) = 721.245, *p *< 0.001; RMSEA = 0.052, CFI = 0.931, TLI = 0.926, SRMR = 0.058]. The *χ*2-difference test between the metric model and the scalar invariance model was significant [*χ*^2^ (16) = 36.826, *p* = 0.002]. Also, the AIC values were the lowest for the model testing metric invariance (AIC_conf_ = 32,199, AIC_metric_ = 32,192, AIC_scalar_ = 32,197. These results indicate metric invariance across gender.

To establish partial scalar invariance the equality constraints on the intercept parameters for three items were sequentially released for items 12 and 13 of the positive social comparison subscale and item 20 of the self-control subscale (see Table 1 for the meaning of these items). The adapted scalar invariance model fit was acceptable [*χ*^2^ (387) = 701.326, *p* < 0.001; RMSEA = 0.050, CFI = 0.935, TLI = 0.929, SRMR = 0.058]. The *χ*^2^-difference test between the metric model and the adapted scalar invariance model was insignificant [*χ*^2^ (13) = 16.907, *p* = 0.204] and the AIC value was also the lowest for the adapted model 2 (AIC = 32,185). Thus, partial scalar invariance for gender was established.

#### 7.2.3. Construct validity

The authentic self-presentation subscale correlated strongly with the item on authenticity of posted positive content (no correlations are reported in the text; all can be found in Table 3). The connectedness subscale was weakly correlated with the Adolescent Friendship Secure Attachment subscale.

**Table 3:** DFSA construct validity and correlations with demographic statistics (study 2).

	Connectedness		Civil participation		Positive social comparison		Authentic self-presentation		Self-control	
	N	r	N	r	N	r	N	r	N	r
Authenticity of posted positive content							854	0.51***		
Secure attachment with a close friend	922	0.10**								
Gender (girls is ref.category)	912	−0.04	730	0.16***	888	.04	831	0.12***	914	−0.04
Age	946	0.02	756	−0.01	921	−0.05	857	0.04	946	−0.04
Age (11–15)	458	0.00	344	−0.06	434	−0.01	412	−0.04	454	−0.03
Age (16–20)	488	0.00	412	0.06	487	0.00	445	0.01	492	0.00
Education track (vocational is ref.cat)	651	0.17***	539	0.19***	637	0.05	595	0.10*	654	−0.02
Education track (vocational)	189	9.08 (2.24)^a^	167	17.62 (3.31)^a^	189	12.67 (2.88)^a^	186	16.63 (3.91)^a^	189	14.46 (2.79)^a^
Education track (technical)	279	9.71 (2.34)^a^	233	18.26 (3.37)^a^	270	12.99 (3.17)^a^	255	16.72 (3.99)^a^	276	14.48 (2.85)^a^
Education track (general)	183	10.16 (2.58)^a^	139	19.38 (3.76)^a^	178	13.10 (3.61)^a^	154	17.73 (3.88)^a^	189	14.34 (3.20)^a^
Education father (high is ref.category)	821	0.03	647	0.07	801	0.05	743	−0.06	822	−0.03
Education father (low)	451	−0.05	365	0.14**	441	0.07	413	0.03	449	0.02
Education father (high)	370	−0.07	283	0.03	360	0.04	330	−0.01	373	−0.02
Education mother (high is ref.category)	877	0.05	694	0.06	854	0.04	794	0.05	876	−0.02
Education mother (low)	328	0.03	263	−0.05	321	−0.05	302	0.01	319	0.00
Education mother (high)	549	−0.02	431	0.02	533	−.03	492	0.05	557	−0.01

Note. *p < 0.05, **p < 0.01, ***p < 0.001.

^a^ Means instead of correlation.

Low education: secondary and lower; high education: post-secondary.

Table 3 shows the correlations between the DFSA scales and demographic variables (i.e., gender, age, educational track, paternal and maternal education level). There was a significant correlation between gender and the civil participation subscale, indicating that the mean score of girls in the civil participation subscale is higher than that of boys. Gender was also significantly correlated with the authentic self-presentation subscale, indicating that girls demonstrate higher scores for authentic self-presentation than boys. Adolescents’ secondary educational track was significantly correlated with the authentic self-presentation subscale, the connectedness subscale, and the civil participation subscale respectively. Adolescents following general education demonstrated higher scores for the three latter subscales than adolescents following professional-technical and vocational education. In all these correlations, higher socioeconomic status signaled higher scores on the digital flourishing dimensions. However, paternal low education was significantly correlated with the civil participation subscale, indicating that adolescents with less-educated fathers demonstrated higher scores on civil participation than the ones with highly educated fathers. No other significant relationships were found (e.g., the relationships between maternal educational status and the DFSA subscales were non-significant).

### 7.3. Brief discussion of study 2

Study 2 finalized the process of validating the scale. The two remaining dimensions were validated with already existent constructs (i.e., authentic self-presentation with the authenticity of posted positive content, and connectedness with secure attachment to a close friend). This study also confirmed the five-dimensional structure of DFSA with a larger sample of adolescents. Furthermore, partial scalar invariance indicates that the majority of the item intercepts do not differ across gender. Thus, DFSA can be used among samples of adolescent boys and girls, as the measurement is invariant across gender.

## 8. General discussion

Scholars have substantially focused more on the negative effects of digital communication than on its positive effects (16). Until the conceptualization of digital flourishing and the development of the Digital Flourishing Scale (DFS), a communication-centered measurement instrument that comprehensively captures the positive perceptions of an individual’s experiences and behaviors in the context of digital communication was absent (13). Research on such an instrument in the context of adolescents’ digital communication use is still lacking. To address this gap, we developed the Digital Flourishing Scale for Adolescents (DFSA). This scale considers the developmental context of adolescence (22) and provides a practical tool for examining adolescents’ perceived flourishing and empowerment when engaging in digital communication.

The current study provides evidence that the 21-item DFSA is a reliable and valid tool that systematically and comprehensively captures digital flourishing in adolescence. The exploratory and confirmatory factor analyses demonstrated the same five-dimensional factor structure of digital flourishing for adolescents and adults (i.e., connectedness, civil participation, positive social comparison, authentic self-presentation, and self-control). Digital flourishing can be investigated as a composite score of all subscales, or its five dimensions can be individually investigated. The subscales can help to inspire research in different subdimensions (a) to examine more positive online behaviors and (b) to complement existing qualitative research with quantitative research. For instance, research on online social comparisons has largely focused on the negative outcomes of these comparison processes in adolescence (37). Our novel subscale may help to foster a new direction of research into the potential positive outcomes of such processes. Similarly, the benefits of self-controlled digital communication have largely been neglected in the field (42). Furthermore, some domains of digital communication have especially been examined qualitatively as no validated scales in adolescents existed to explore these subjects quantitatively. One of these domains is the subfield of online authentic self-presentation, which for now has especially been qualitatively studied in adolescents (32–34).

The results demonstrated the construct validity of the DFSA. The five flourishing dimensions were significantly associated with one or more scales for the satisfaction of basic psychological needs in adolescence (25). Moreover, the five flourishing dimensions were validated by showing significant relationships with related constructs of digital communication in Study 1 and Study 2 (e.g., a negative relationship occurred between Internet aggression and civil participation).

Further, DFSA was found to be largely invariant across genders. Future research should take into account that three items were found to differ between boys and girls (“Seeing how others present themselves online motivates me to make changes in my own life.”, “Comparing myself to others online motivates me to accomplish the things I want in life.”, and “When I browse through online content, I feel in control of how I spend my time.”), as only partial scalar invariance was established after eliminating variance in these three items across gender.

Both studies demonstrated high mean scores for digital flourishing in the adolescents’ samples. On average, adolescents scored the highest on civil participation, followed by self-control, authentic self-presentation, positive social comparison, and connectedness. Since the DFSA is a self-reported measure, adolescents could have assigned higher scores for more socially desirable online behaviors, such as civil participation. Thus, the results also potentially reflect adolescents’ norms of digital flourishing practices (69).

Despite the overall high mean scores for digital flourishing, the results also indicated that not all groups of adolescents thrive online equally. First, gender differences were found in DFSA. Girls scored higher on civil participation than boys. This finding supports previous literature, as girls have been reported to score higher than boys for respect and civic engagement online (41). The potential explanations for our results could be that girls are likely to be socialized to be more “kind” than boys and to act prosocially more often than boys (70). In contrast with earlier qualitative findings (35), girls demonstrated significantly higher scores on authentic self-presentation than boys in Study 2. Girls attach more importance to self-presentation than boys (35). Moreover, recent literature indicates that authentic self-presentation is nowadays considered normative (32). Potentially, the normative expectancy to present oneself as authentic, combined with the higher importance attached to self-presentation by girls, explains the reported gender difference.

Second, our study offers some initial insights into the role of socioeconomic status (SES) in digital flourishing. According to the digital divide literature, adolescents with a lower SES exhibit a significantly lower level of digital skills and outcomes (71). Adolescents’ SES can be predicted by parental education level (72), which is strongly related to the secondary track choice of adolescents (73). Correlations between the adolescents’ education track and several digital flourishing subscales in Study 2 (and in Study 1 to some extent) demonstrated that the mean scores for civil participation, authentic self-presentation, and connectedness were significantly lower among adolescents in vocational and professional-technical education than among adolescents in the general education track. Meanwhile, adolescents with less-educated fathers demonstrated significantly higher scores on positive social comparisons than adolescents with highly educated fathers. The results regarding mother’s education were surprisingly not significant. These findings highlight that further research is needed on how parental educational status relates to digital flourishing across adolescents. On average, mothers and fathers report similar levels of digital skills though in different domains (e.g., mothers report more advanced skills in information search and in privacy management but fathers report more advanced skills in coding and content editing) (74). Potentially, mothers and fathers might also differ in their digital flourishing skills and communicate these skills to their children differently according to their SES. Research may further examine this reasoning. Additionally, the digital divide literature has typically focused on the negative outcomes of information and communication technology use. Our newly developed scale will help research how disadvantaged adolescents lack empowered digital communication and the positive outcomes these digital communication skills bring (71).

Moreover, the DFSA focuses on digital communication in general. Further studies could adjust the scale (or its subscales) to refer to different technological contexts (e.g., different social media platforms such as Instagram vs. Snapchat) (13). Such research may explore whether users’ digital flourishing skills may differ depending on different digital communication tools.

## 9. Limitations

Although Study 1 and Study 2 supported the reliability and validity of the scale, they had some limitations. First, in Study 1, the sample of adolescents was rather small (*N *= 147), and the validity measures were divided into and distributed among two sub-samples. The low sample size can be explained by the COVID-19 pandemic and the period of data collection (i.e., at the end of the school year, adolescents’ motivation to participate was low, and access to participants through schools was limited). Second, the sample used in Study 1 primarily included adolescents between 16 and 19 years of age. These participants also mainly followed a general education track and originated from families with a high SES. Study 2 covered a more diverse sample (*N* = 1,046) spanning a larger age range (11–18 years) and including more adolescents from different educational tracks and families with diverse SES. Third, Studies 1 and 2 were conducted using Slovenian samples, which limits the representativeness of the findings to other cultures. Additional research is needed to further validate the scale. The DFSA should be employed in different countries and languages. Such cross-cultural validation could allow for a wider application of the scale.

Finally, similar to the bulk of quantitative measurements, DFSA is a self-reported measure that offers insights into adolescents’ perceptions of their experiences and behaviors in digital communication. Therefore, it is likely that adolescents provide socially desirable responses (69). Future studies should consider using a combination of self-reporting and more “objective” tools that measure adolescents’ digital behavior (75) (e.g., coding actual online posts).

## Data Availability

The datasets presented in this study can be found in online repositories. The names of the repository/repositories and accession number(s) can be found below: https://osf.io/9wuyb/?view_only=9e64aa7358ed40a0823a8cb75c49c3ae.

## Footnotes

^1^More information about the project can be found on the website: https://www.projectmimic.eu/.
