# Supplementary material for: Positive digital communication among youth: The development and validation of the digital flourishing scale for adolescents
Source: Front Digit Health. 2022 Sep 1;4:975557. doi: 10.3389/fdgth.2022.975557 (PMC9474732; doi:10.3389/fdgth.2022.975557)
Supplement: Supplementary file 1 [file Table_1.pdf]

Appendix A: Table: The adaptation of DFS to DFSA

Instruction

**DFS**
The following questions are all concerned with learning more about your online communication habits and experiences. Again, we refer to online communication to include professional and personal online communication across a variety of online platforms, including, but not limited to email, texting, direct messaging, interacting on social media, phone conversations, facetime, zoom ect.<br>There is no right or wrong answer. We are just interested in your most truthful opinions. Please indicate how much you disagree or agree with each statement.<br><br>Strongly disagree (1) – Strongly agree (7)
**DFS for Adolescents**
The following questions are all concerned with learning more about your online communication habits and experiences during the past month. We refer to online communication to include conversations and participation across a variety of online applications. Examples of online communication are emailing, chatting (e.g., smartphone SMS texting, Direct messaging tools like WhatsApp, Viber), interacting on social media (e.g., posting, commenting or liking posts on SnapChat, Instagram, TikTok or YouTube), phone conversations, FaceTime, zoom etc.<br>There is no right or wrong answer. We are just interested in your most truthful opinions. **Please indicate how true the following behaviors were for you in the past month using the options from "Not at all true of me" to "Very true of me". If you never engaged in one of the online communication behaviors listed below, please select "Not applicable to me".**
**DFS for Adolescents SLO**
Z naslednjimi vprašanji želimo izvedeti več o tvojih navadah in izkušnjah s komuniciranjem preko spleta v zadnjem mesecu. Z izrazom »komuniciranje preko spleta« mislimo na pogovore in udeležbo na različnih spletnih aplikacijah. Na primer na e-pošto, klepetanje (npr. s SMS sporočili, z neposrednimi sporočili npr. preko Whatsappa, Viberja), komunikacijo na družbenih omrežjih (npr. objavljanje, komentiranje ali všečkanje objav na SnapChatu, Instagramu, TikToku, Youtube-u), pa tudi telefonske pogovore, FaceTime, Zoom in drugo.<br><br>Ni pravilnih ali napačnih odgovorov, zanima nas le tvoje iskreno mnenje.<br><br>**Prosimo, oceni, v kolikšni meri je v zadnjem mesecu vsaka trditev veljala zate, tako da izbereš eno izmed možnosti od zame »Sploh ne drži« do »Popolnoma drži«. Če katerega od omenjenih vedenj na spletu nisi počel/-a, izberi možnost »Zame ni relevantno«.**<br><br>Not at all true of me (1), Not true of me (2), Partly not true, partly true of me (3), True of me (4), Very true of me (5), Not applicable to me (99)

Items per DF subscale used for Study 1

DF Subscale	DFS Adolescents	DFS Adolescent SLO
**Connectedness**		
The following statements are about your experiences with connecting with others online. When assessing the statements think about the last month. Naslednje trditve se nanašajo na tvoje izkušnje glede povezovanja z drugimi na spletu. Ko ocenjuješ spodnje trditve, imej v mislih zadnji mesec.		
1. I feel part of a community when I interact with others online	1. I feel part of a group when I communicate with others online.	Ko komuniciram z drugimi preko spleta, se počutim kot del skupine na spletu.
2. I feel a bond to my online community/network	2. I feel closely connected to the groups I connect with online.	Počutim se tesno povezanega/-o s skupinami, s katerimi se družim na spletu.
3. I find great purpose and meaning in my online interactions	3. I find my online communication (e.g. chatting with peers, playing online games with others) very important.	Zdi se mi, da je moje komuniciranje preko spleta (npr. pogovori s prijatelji, igranje iger z drugimi preko spleta) zame zelo pomembno.
4. When I browse through my online communication platforms, I feel a sense of connection to others	4. When I browse (scroll) through my online apps (like Snapchat, TikTok, Instagram etc.), I feel connected to others.	Ko brskam (»scrollam«) po spletnih aplikacijah (kot so npr. Snapchat, TikTok, Instagram), se počutim povezan/-a z drugimi.
5. I could turn to my online community if I needed specific advice on how to handle a problem	5. I could turn to people who I connect with online (e.g. acquaintances) if I needed advice on a problem.	Če potrebujem nasvet glede problema, se lahko obrnem na osebe, s katerimi se družim preko spleta (npr. znance).
**DF Subscale**	**DFS Adolescents**	**DFS Adolescent SLO**
**Civil participation**		
The following statements are about how you express your opinion online. When assessing the statements think about the last month. Naslednje trditve se nanašajo na to, kako si izražal/-a svoje mnenje na spletu. Ko ocenjuješ spodnje trditve, imej v mislih zadnji mesec.		
1. When I interact with others about politics online, I know how to have a civil discussion	When I talk to others online about politics (e.g. about the government, the President, elections), I know how to do it politely.	Ko se pogovarjam z drugimi preko spleta o politiki (npr. o vladi, predsedniku, volitvah, …), znam pri tem biti spoštljiv/-a.
3. During my interactions online, I know how to take a stand for something important to me in a civil manner	When I talk to others online about something important to me, I know how to stand up for it in a polite manner.	Ko se pogovarjam z drugimi preko spleta o nečem, kar mi veliko pomeni, znam na spoštljiv način to tudi zagovarjati.

4. In online discussions, I know how to get my point across without offending people	When I talk to others online, I know how to share my point of view without offending them.	Ko se pogovarjam z drugimi preko spleta, znam izraziti mnenje, brez da sem pri tem žaljiv/-a.
6. When I interact with others online I tend to respond reasonably even when they make me angry	When something that others say or do online make me feel angry, I am able to respond in a calm way.	Kadar drugi na spletu rečejo ali storijo nekaj, kar me razjezi, sem se na to zmožen/-na odzvati na miren način.
7. When I communicate online, I am careful to adapt my comments and behaviors to be appropriate for my audience	When I communicate online, I am careful to adapt my comments and behaviors to be appropriate for whoever will read them (e.g. my friends, my teacher, my parents, younger children).	Kadar komuniciram preko spleta, pazim, da prilagodim svoje komentarje in vedenje na način, da so primerni za vse, ki bi jih prebrali (npr. moji prijatelji, učitelji, starši, mlajši otroci).
**DF Subscale**	**DFS Adolescents**	**DFS Adolescent SLO**
**Positive social comparison**		
The following statements are about comparing yourself to others on social media or other online applications. When assessing the statements think about the last month. Naslednje trditve se nanašajo na to, kako si se primerjal/-a z drugimi na družbenih omrežjih in drugih spletnih aplikacijah. Ko ocenjuješ spodnje trditve, imej v mislih zadnji mesec.		
1. Comparing myself to others online motivates me to accomplish my goals	Comparing myself to others online motivates me to accomplish the things I want in life.	To, da se primerjam z drugimi na spletu, me motivira, da dosežem stvari, ki jih želim v življenju.
2. I compare my life to those online who I can learn from	I compare my life to those people online (e.g. peers, influencers) who I can learn from.	Na spletu svoje življenje primerjam s tistimi ljudmi (npr. sovrstniki, vplivneži), od katerih se lahko česa naučim.
4. Seeing how others present themselves online motivates me to make changes in my own life	Seeing how others present themselves online motivates me to make changes in my own life.	To, da vidim, kako se drugi predstavljajo na spletu, me motivira, da sprejmem spremembe v svojem življenju.
6. I compare my life to those online who are going to push me to be better	I compare my life to those people online (e.g. peers, influencers) who are going to push me to be better.	Na spletu primerjam svoje življenje s tistimi ljudmi (npr. sovrstniki, vplivneži), ki me spodbudijo, da sem boljši/-a.
7. Seeing other's achievements online inspires me to do better	Seeing others' achievements online inspires me to do better.	Dosežki drugih, ki jih vidim na spletu, me navdihnejo, da postanem boljši/-a.
**DF Subscale**	**DFS Adolescents**	**DFS Adolescent SLO**
**Authentic Self-Presentation**		
The following statements are about how you present yourself online. When assessing the statements think about the last month.		

Naslednje trditve se nanašajo na to, kako si se predstavljal/-a drugim na spletu. Ko ocenjuješ spodnje trditve, imej v mislih zadnji mesec.		
1. I allow my social network to see who I really am	I allow people who I connect with online to see who I really am.	Ljudem, s katerimi se družim preko spleta, dovolim, da me vidijo takega/-o kot resnično sem.
2. I feel comfortable presenting who I truly am online, in the same way I do offline	I feel comfortable presenting who I truly am online, in the same way I do offline.	Na spletu brez zadržkov pokažem pravega/-o sebe, na enak način kot to storim v resničnem življenju.
3. What I post online reflects who I really am	What I post online reflects who I really am.	Kar objavim na spletu, odraža to, kdo jaz v resnici sem.
5. I show my true self online	I show my true self online.	Na spletu pokažem pravega/-o sebe.
7. When interacting online, I feel comfortable presenting the person I am	When communicating online, I feel comfortable presenting the person I am.	Ko komuniciram preko spleta, se brez zadržkov predstavim takega/-o kot sem.
**DF Subscale**	**DFS Adolescents**	**DFS Adolescent SLO**
**Self-control**		
The following statements are about the control you have to have about online communication. When assessing the statements think about the last month. Naslednje trditve se nanašajo na nadzor tvojega komuniciranja na spletu. Ko ocenjuješ spodnje trditve, imej v mislih zadnji mesec.		
1. For the most part, I feel in control of how much time I spend interacting with others online	For the most part, I feel in control of how much time I spend communicating with others online (e.g. chatting with friends, posting on Instagram, playing online games with others).	Zdi se mi, da večinoma lahko nadzorujem, koliko časa porabim za komuniciranje preko spleta z drugimi (npr. klepetanje s prijatelji, objavljanje prispevkov na Instagramu, igranje igric z drugimi preko spleta).
2. I feel in control over when to start and when to stop spending time on online communication	I feel in control of when to start and when to stop spending time on online communication.	Zdi se mi, da lahko nadzorujem, kdaj začnem in kdaj preneham s komuniciranjem preko spleta.
3. I communicate online when I want to, not when notifications tell me to	I communicate online when I want to, not when notifications tell me to.	Preko spleta komuniciram, ko to sam/-a želim, ne pa, ko mi to narekujejo obvestila na napravah in v aplikacijah.
6. I am able to disconnect from my online interactions when I need a break	I am able to disconnect from my online communication when I need a break.	Zmožen/-na sem se odklopiti od svojega komuniciranja preko spleta, kadar potrebujem odmor.
7. When I browse through content online I feel in	When I browse through online content I feel in	Ko brskam po vsebinah na spletu, se mi zdi, da imam

control of how I spend my time	control of how I spend my time.	nadzor nad tem, kako preživljam svoj čas.
