# Supplementary material for: Positive digital communication among youth: The development and validation of the digital flourishing scale for adolescents
Source: Front Digit Health. 2022 Sep 1;4:975557. doi: 10.3389/fdgth.2022.975557 (PMC9474732; doi:10.3389/fdgth.2022.975557)
Supplement: Supplementary file 2 [file Table_2.pdf]

DFSA scale and scales to measure construct validity Study 1

Appendix B: DFSA Scale in English (Study 1)

The following questions are all concerned with learning more about your online communication habits and experiences during the past month. We refer to online communication to include conversations and participation across a variety of online applications. Examples of online communication are emailing, chatting (e.g., smartphone SMS texting, Direct messaging tools like WhatsApp, Viber), interacting on social media (e.g., posting, commenting or liking posts on SnapChat, Instagram, TikTok or YouTube), phone conversations, FaceTime, zoom etc.

There is no right or wrong answer. We are just interested in your most truthful opinions.

Please indicate how true the following behaviors were for you in the past month using the options from "Not at all true of me" to "Very true of me". If you never engaged in one of the online communication behaviors listed below, please select "Not applicable to me".

The following statements are about your experiences with connecting with others online. When assessing the statements think about the past month.

	Not at all true of me	Not true of me	Partly not true, partly true of me	True of me	Very true of me	Not applicable to me
When I browse (scroll) through my online apps (like Snapchat, TikTok, Instagram etc.), I feel connected to others.	☐	☐	☐	☐	☐	☐
I feel part of a group when I communicate with others online.	☐	☐	☐	☐	☐	☐
I find my online communication (e.g., chatting with peers, playing online games with others) very important.	☐	☐	☐	☐	☐	☐
I could turn to people who I connect with online (e.g., acquaintances) if I needed advice on a problem.	☐	☐	☐	☐	☐	☐
I feel closely connected to the groups I connect with online.	☐	☐	☐	☐	☐	☐

The following statements are about how you express your opinion online. When assessing the statements think about the past month.

	Not at all true of me	Not true of me	Partly not true, partly true of me	True of me	Very true of me	Not applicable to me

When I talk to others online, I know how to share my point of view without offending them.	☐	☐	☐	☐	☐	☐
When I communicate online, I am careful to adapt my comments and behaviors to be appropriate for whoever will read them (e.g., my friends, my teacher, my parents, younger children).	☐	☐	☐	☐	☐	☐
When I talk to others online about something important to me, I know how to stand up for it in a polite manner.	☐	☐	☐	☐	☐	☐
When I talk to others online about politics (e.g., about the government, the President, elections), I know how to do it politely.	☐	☐	☐	☐	☐	☐
When something that others say or do online makes me feel angry, I am able to respond in a calm way.	☐	☐	☐	☐	☐	☐

The following statements are about comparing yourself to others on social media or other online applications. When assessing the statements think about the past month.

	Not at all true of me	Not true of me	Partly not true, partly true of me	True of me	Very true of me	Not applicable to me
Seeing others' achievements online inspires me to do better.	☐	☐	☐	☐	☐	☐
Seeing how others present themselves online motivates me to make changes in my own life.	☐	☐	☐	☐	☐	☐
I compare my life to those people online (e.g., peers, influencers) who I can learn from.	☐	☐	☐	☐	☐	☐
Comparing myself to others online motivates me to accomplish the things I want in life.	☐	☐	☐	☐	☐	☐
I compare my life to those people online (e.g., peers, influencers) who are going to push me to be better.	☐	☐	☐	☐	☐	☐

The following statements are about how you present yourself online. When assessing the statements think about the past month.

	Not at all true of me	Not true of me	Partly not true, partly true of me	True of me	Very true of me	Not applicable to me
I show my true self online.	☐	☐	☐	☐	☐	☐
When communicating online, I feel comfortable presenting the person I am.	☐	☐	☐	☐	☐	☐
What I post online reflects who I really am.	☐	☐	☐	☐	☐	☐
I feel comfortable presenting who I truly am online, in the same way I do offline.	☐	☐	☐	☐	☐	☐
I allow people who I connect with online to see who I really am.	☐	☐	☐	☐	☐	☐

The following statements are about the control you have to have about online communication. When assessing the statements think about the past month.

	Not at all true of me	Not true of me	Partly not true, partly true of me	True of me	Very true of me	Not applicable to me
I feel in control of when to start and when to stop spending time on online communication.	☐	☐	☐	☐	☐	☐
For the most part, I feel in control of how much time I spend communicating with others online (e.g., chatting with friends, posting on Instagram, playing online games with others).	☐	☐	☐	☐	☐	☐
I communicate online when I want to, not when notifications tell me to.	☐	☐	☐	☐	☐	☐
When I browse through online content I feel in control of how I spend my time.	☐	☐	☐	☐	☐	☐
I am able to disconnect from my online communication when I need a break.	☐	☐	☐	☐	☐	☐

Appendix B: DFSA Scale in Slovenian (Study 1)

Z naslednjimi vprašanji želimo izvedeti več o tvojih navadah in izkušnjah s komuniciranjem preko spleta v zadnjem mesecu. Z izrazom »komuniciranje preko spleta« mislimo na pogovore in udeležbo na različnih spletnih aplikacijah. Na primer na e-pošto, klepetanje (npr. s SMS sporočili, z neposrednimi sporočili npr. preko Whatsappa), komunikacijo na družbenih omrežjih (npr. objavljanje, komentiranje ali všečkanje objav na SnapChatu, Instagramu, TikToku, Youtube-u), pa tudi telefonske pogovore, FaceTime, Zoom in drugo.

Ni pravilnih ali napačnih odgovorov, zanima nas le tvoje iskreno mnenje.

Prosimo, oceni, v kolikšni meri je v zadnjem mesecu vsaka trditev veljala zate, tako da izbereš eno izmed možnosti od zame »Sploh ne drži« do »Popolnoma drži«. Če katerega od omenjenih vedenj na spletu nisi počel/-a, izberi možnost »Zame ni relevantno«.

Naslednje trditve se nanašajo na tvoje izkušnje glede povezovanja z drugimi na spletu. Ko ocenjuješ spodnje trditve, imej v mislih zadnji mesec.

	Sploh ne drži	Ne drži	Deloma ne drži deloma drži	Drži	Popolnoma drži	Zame ni relevantno
Ko brskam (»scrollam«) po spletnih aplikacijah (kot so npr. Snapchat, TikTok, Instagram), se počutim povezan/-a z drugimi.	☐	☐	☐	☐	☐	☐
Ko komuniciram z drugimi preko spleta, se počutim kot del skupine na spletu.	☐	☐	☐	☐	☐	☐
Zdi se mi, da je moje komuniciranje preko spleta (npr. pogovori s prijatelji, igranje iger z drugimi preko spleta) zame zelo pomembno.	☐	☐	☐	☐	☐	☐
Če potrebujem nasvet glede problema, se lahko obrnem na osebe, s katerimi se družim preko spleta (npr. znance).	☐	☐	☐	☐	☐	☐
Počutim se tesno povezanega/-o s skupinami, s katerimi se družim na spletu.	☐	☐	☐	☐	☐	☐

Naslednje trditve se nanašajo na to, kako si izražal/-a svoje mnenje na spletu. Ko ocenjuješ spodnje trditve, imej v mislih zadnji mesec.

	Sploh ne drži	Ne drži	Deloma ne drži deloma drži	Drži	Popolnoma drži	Zame ni relevantno

Ko se pogovarjam z drugimi preko spleta, znam izraziti mnenje, brez da sem pri tem žaljiv/-a.	☐	☐	☐	☐	☐	☐
Kadar komuniciram preko spleta, pazim, da prilagodim svoje komentarje in vedenje na način, da so primerni za vse, ki bi jih prebrali (npr. moji prijatelji, učitelji, starši, mlajši otroci).	☐	☐	☐	☐	☐	☐
Ko se pogovarjam z drugimi preko spleta o nečem, kar mi veliko pomeni, znam na spoštljiv način to tudi zagovarjati.	☐	☐	☐	☐	☐	☐
Ko se pogovarjam z drugimi preko spleta o politiki (npr. o vladi, predsedniku, volitvah, …) znam pri tem biti spoštljiv/-a.	☐	☐	☐	☐	☐	☐
Kadar drugi na spletu rečejo ali storijo nekaj, kar me razjezi, sem se na to zmožen/-na odzvati na miren način.	☐	☐	☐	☐	☐	☐

Naslednje trditve se nanašajo na to, kako si se primerjal/-a z drugimi na družbenih omrežjih in drugih spletnih aplikacijah. Ko ocenjuješ spodnje trditve, imej v mislih zadnji mesec.

	Sploh ne drži	Ne drži	Deloma ne drži deloma drži	Drži	Popolnoma drži	Zame ni relevantno
Dosežki drugih, ki jih vidim na spletu, me navdihnejo, da postanem boljši/-a.	☐	☐	☐	☐	☐	☐
To, da vidim, kako se drugi predstavljajo na spletu, me motivira, da sprejmem spremembe v svojem življenju.	☐	☐	☐	☐	☐	☐
Na spletu svoje življenje primerjam s tistimi ljudmi (npr. sovrstniki, vplivneži), od katerih se lahko česa naučim.	☐	☐	☐	☐	☐	☐

To, da se primerjam z drugimi na spletu, me motivira, da dosežem stvari, ki jih želim v življenju.	☐	☐	☐	☐	☐	☐
Na spletu primerjam svoje življenje s tistimi ljudmi (npr. sovrstniki, vplivneži), ki me spodbudijo, da sem boljši/-a.	☐	☐	☐	☐	☐	☐

Naslednje trditve se nanašajo na to, kako si se predstavljal/-a drugim na spletu. Ko ocenjuješ spodnje trditve, imej v mislih zadnji mesec.

	Sploh ne drži	Ne drži	Deloma ne drži deloma drži	Drži	Popolnoma drži	Zame ni relevantno
Na spletu pokažem pravega/-o sebe.	☐	☐	☐	☐	☐	☐
Ko komuniciram preko spleta, se brez zadržkov predstavim takega/-o kot sem.	☐	☐	☐	☐	☐	☐
Kar objavim na spletu, odraža to, kdo jaz v resnici sem.	☐	☐	☐	☐	☐	☐
Na spletu brez zadržkov pokažem pravega/-o sebe, na enak način kot to storim v resničnem življenju.	☐	☐	☐	☐	☐	☐
Ljudem, s katerimi se družim preko spleta, dovolim, da me vidijo takega/-o kot resnično sem.	☐	☐	☐	☐	☐	☐

Naslednje trditve se nanašajo na nadzor tvojega komuniciranja na spletu. Ko ocenjuješ spodnje trditve, imej v mislih zadnji mesec.

	Sploh ne drži	Ne drži	Deloma ne drži deloma drži	Drži	Popolnoma drži	Zame ni relevantno
Zdi se mi, da lahko nadzorujem, kdaj začnem in kdaj preneham s komuniciranjem preko spleta.	☐	☐	☐	☐	☐	☐

Zdi se mi, da večinoma lahko nadzorujem, koliko časa porabim za komuniciranje preko spleta z drugimi (npr. klepetanje s prijatelji, objavljanje prispevkov na Instagramu, igranje igric z drugimi preko spleta).	☐	☐	☐	☐	☐	☐
Preko spleta komuniciram, ko to sam/-a želim, ne pa, ko mi to narekujejo obvestila na napravah in v aplikacijah.	☐	☐	☐	☐	☐	☐
Ko brskam po vsebinah na spletu, se mi zdi, da imam nadzor nad tem, kako preživljam svoj čas.	☐	☐	☐	☐	☐	☐
Zmožen/-na sem se odklopiti od svojega komuniciranja preko spleta, kadar potrebujem odmor.	☐	☐	☐	☐	☐	☐

Appendix B: Scales used to estimate construct validity in English (Study 1)

A) DEMOGRAPHIC VARIABLES

1. What is your **birthday**? Year ...

(dropdown 2001-2009)

2. What is the name of your school?

☐ Gimnazija in srednja šola Kočevje (1)
☐ Srednja šola Veno Pilon Ajdovščina (2)
☐ Gimnazija Tolmin (3)
☐ Srednja gozdarska in lesarska šola Postojna (4)
☐ Prva gimnazija Maribor (5)
☐ Other, specify (6): _____________

3. What **class** are you in?:

☐1. letnik secondary school
☐2. letnik secondary school
☐3. letnik secondary school
☐4. letnik secondary school
☐9. razred primary school
☐8. razred primary school
☐7. razred primary school
☐6. razred primary school
☐Drugo (vpiši): _______

4. Which **educational tract** do you follow?

☐ Secondary vocational education (e.g. carpenter, merchant, metalworker – toolmaker)
☐ Secondary professional-technical education (e.g. economic technician, mechanical technician, pre-school teacher)
☐ Secondary general education (e.g. general high school, economic high school)

5. I am a:

☐ Boy
☐ Girl
☐ Other
☐ Prefer not to say

The next questions are about you and your family.

6. What is the highest degree that **your father** obtained?

☐ Unfinished primary education
☐ Primary Education
☐ Secondary Education
☐ Higher vocational education
☐ Professional higher education and University (Bachelor, Master’s, PhD)

☐ I don’t know, but my dad works as: ___
☐ I can’t respond

7. What is the highest degree that **your mother** obtained?

☐ Unfinished primary education
☐ Primary Education
☐ Secondary Education
☐ Higher vocational education
☐ Professional higher education and University (Bachelor, Master’s, PhD)
☐ I don’t know, but my dad works as: ___
☐ I can’t respond

8. What is your **ethnic** background? You can choose multiple options

☐ West European (e.g. Belgium, France, United Kingdom, The Netherlands)
☐ Central European (e.g. Germany, Austria, Slovenia, Poland, Hungary)
☐ East European (e.g. Russia, Ukraine, Belarus)
☐ South European (e.g. Italy, Spain, Portugal)
☐ South-east European (e.g. Croatia, Serbia, North Macedonia, Bulgaria, Romania)
☐ North European (e.g. Sweden, Norway, Finland)
☐ African or Middle-East (e.g. Morocco, Egypt, Congo…)
☐ North-American (Canada or USA)
☐ South-American or Latin American (e.g. Cuba, Mexico, Brazil…)
☐ Asian (e.g. China, India, Japan, Cambodia, Pakistan, …)
☐ Romani
☐ Other, specify: ……………………………………………………
☐ I don’t know

B) Technoference (Stockdale, Coyne, Padilla-Walker, 2018)

9. How often did you use your smartphone while talking to a friend in the past month?
   - Never
   - Rarely
   - Sometimes
   - Often
   - Always

10. Below are some statements about using mobile phones during conversations with your friends. For each statement, please indicate how true or not true the following behaviors are for you. When assessing the statements think about the past month.

	Not at all true of me	Not true of me	Partly not true, partly true of me	True of me	Very true of me
I ignore my friends when I am on my cell phone	☐	☐	☐	☐	☐
My friends struggle to get my attention when I am on my cell phone	☐	☐	☐	☐	☐

I check my cell phone even if I'm right in the middle of a conversation with my friends.	☐	☐	☐	☐	☐

<u>The following questions are about your social media use.</u>
<u>If you don’t use social media, please select “not applicable for me”.</u>

C) Brief Measure of Social Media Self Control Failure (Du et al., 2018)

11. How often in the past month did you give in to a desire to use social media even though your social media use at that particular moment ...

	Never	Rarely (1–3 times)	Sometimes (4–6 times)	Often (7–9 times)	Very often (10 or more times)	Not applicable to me
… conflicted with other goals (e.g. doing things for school or other tasks)?	☐	☐	☐	☐	☐	☐
… made you use your time less efficiently?	☐	☐	☐	☐	☐	☐
… made you delay other things you want or need to do?	☐	☐	☐	☐	☐	☐

D) Social media-induced inspiration scale (Meier & Schäfer, 2018)

12. The following questions are about how you felt when you used social media in the past month. For each statement please indicate the extent to which you agree or disagree that the following feelings apply to you. If you don’t use social media, please select “not applicable to me”.

	Strongly disagree	Disagree	Partly disagree, partly agree	Agree	Strongly agree	Not applicable to me
When I use social media I experience inspiration.	☐	☐	☐	☐	☐	☐
When I use social media I am inspired by the posts of other users to do something [new].	☐	☐	☐	☐	☐	☐

E) Posting positive social media content (short form) (Schreurs & Vandenbosch 2021)

13. The following questions are about how you presented yourself when you used social media in the past month. For each statement please indicate how often the following behavior applies to you from never to very often. If you don't use social media, please select "not applicable to me".

How often do you post on most public applications such as social media posts in which you …

	Never	Rarely	Sometim es	Often	Very often	Not applicabl e to me
... look beautiful.	☐	☐	☐	☐	☐	☐
... show that have a lot of fun.	☐	☐	☐	☐	☐	☐
... look successful (e.g., you achieve something at school or in a hobby).	☐	☐	☐	☐	☐	☐
... show how great a friendship is (e.g., that it is clear you have much fun together).	☐	☐	☐	☐	☐	☐
... do nice things (e.g., go to the movie theaters or to the zoo, have something to drink/eat with a friend).	☐	☐	☐	☐	☐	☐
... show a nice clothing style.	☐	☐	☐	☐	☐	☐
... show that you are happy.	☐	☐	☐	☐	☐	☐
... show a nice holiday you have done.	☐	☐	☐	☐	☐	☐

F) Internet aggression (Werner et al.2010)

14. The following statements are about your behavior on the Internet (e.g. on social media). Please, indicate how often in the previous month you engaged in the following behaviors from never to 5 or more times. If you don't use Internet, please select "not applicable to me".

	Never	1–2 times	3–4 times	5 or more times	Not applicable to me
I used the Internet to threaten or embarrass someone (e.g., by posting or sending messages about them for other people to see).	☐	☐	☐	☐	☐

I told others to block instant messages from someone I didn't like or I was mad at.	☐	☐	☐	☐	☐
I used the Internet to play a joke or annoy someone I was mad at.	☐	☐	☐	☐	☐
I made rude or nasty comments about someone else online.	☐	☐	☐	☐	☐

<u>The next questions are about your life and feelings.</u>

G) The satisfaction of basic psychological needs in adolescents (Girelli et al 2019)

15. Read the following sentences carefully and think about your life. Please, indicate how true each statement is for you from "Not at all true of me" to "Very true of me".

	Not at all true of me	Not true of me	Partly not true, partly true of me	True of me	Very true of me
I feel free to decide for myself how to do my things.	☐	☐	☐	☐	☐
I feel good at doing many things.	☐	☐	☐	☐	☐
I like the people I interact with.	☐	☐	☐	☐	☐
I feel like I am free to decide for myself how to live my life.	☐	☐	☐	☐	☐
I am able to learn interesting new skills.	☐	☐	☐	☐	☐
I get along with people I meet.	☐	☐	☐	☐	☐
Generally, I feel free to express what I really think.	☐	☐	☐	☐	☐
I get much of a chance to show how capable I am.	☐	☐	☐	☐	☐
I consider the people I regularly interact with to be my friends.	☐	☐	☐	☐	☐
I feel like I can pretty much be myself.	☐	☐	☐	☐	☐

People tell me that I am good at what I do.	☐	☐	☐	☐	☐
People are generally pretty friendly towards me.	☐	☐	☐	☐	☐

Appendix B: Scales used to estimate construct validity in Slovenian (Study 1)

A) DEMOGRAPHIC VARIABLES

1. Kdaj si rojen/-a? Leta …….

(dropdown 2001-2009)

2. Katero šolo obiskuješ?

☐ Gimnazija in srednja šola Kočevje (1)
☐ Srednja šola Veno Pilon Ajdovščina (2)
☐ Gimnazija Tolmin (3)
☐ Srednja gozdarska in lesarska šola Postojna (4)
☐ Prva gimnazija Maribor (5)
☐ Drugo (vpiši): (6) ____

3. V katerem **letniku** si?:

☐1. letnik SŠ
☐2. letnik SŠ
☐3. letnik SŠ
☐4. letnik SŠ
☐9. razred OŠ
☐8. razred OŠ
☐7. razred OŠ
☐6. razred OŠ
☐Drugo (vpiši): _______

4. V kateri **program izobraževanja** si vpisan/-a?

☐ Srednje poklicno izobraževanje (npr. mizar, trgovec, oblikovalec kovin – orodjar)
☐ Srednje strokovno-tehniško izobraževanje (npr. ekonomski tehnik, strojni tehnik, vzgojitelj predšolskih otrok)
☐ Srednje splošno izobraževanje (npr. gimnazija, ekonomska gimnazija)

5. Sem:

☐ Fant
☐ Dekle
☐ Drugo
☐ Ne želim odgovoriti

Naslednja vprašanja so o tebi in tvoji družini.

6. Katera je najvišja dosežena izobrazba tvojega **očeta?**

☐ Nedokončana osnovnošolska izobrazba
☐ Osnovnošolska izobrazba
☐ Srednješolska izobrazba
☐ Višješolska izobrazba
☐ Visokošolska in univerzitetna izobrazba (diploma, magisterij, doktorat)

☐ Ne vem, ampak moj oče je zaposlen kot _____
☐ Ne morem odgovoriti

7. Katera je najvišja dosežena izobrazba tvoje **mame?**

☐ Nedokončana osnovnošolska izobrazba
☐ Osnovnošolska izobrazba
☐ Srednješolska izobrazba
☐ Višješolska izobrazba
☐ Visokošolska in univerzitetna izobrazba (diploma, magisterij, doktorat)
☐ Ne vem, ampak moja mama je zaposlena kot _____
☐ Ne morem odgovoriti

8. Kako bi opisal svojo etnično pripadnost? Izbereš lahko več odgovorov.

☐ Zahodni/-a Evropejec/-ka (npr. Belgija, Francija, Velika Britanija, Nizozemska …)
☐ Srednji/-a Evropejec/-ka (npr. Nemčija, Avstrija, Slovenija, Poljska, Madžarska …)
☐ Vzhodni/-a Evropejec/-ka (npr. Rusija, Ukrajina, Belorusija …)
☐ Južni/-a Evropejec/-ka (npr. Italija, Španija, Portugalska …)
☐ Jugovzhodni/-a Evropejec/-ka (npr. Hrvaška, Srbija, Severna Makedonija, Bolgarija, Romunija …)
☐ Severni/-a Evropejec/-ka (npr. Švedska, Norveška, Finska …)
☐ Afričan/-ka ali prebivalec/-ka Bližnjega vzhoda (npr. Maroko, Egipt, Kongo …)
☐ Severnoameričan/-ka (Kanada ali Združene države Amerike)
☐ Južnoameričan/-ka ali prebivalec/-ka Latinske Amerike (npr. Kuba, Mehika, Brazilija …)
☐ Azijec/-ka (npr. Kitajska, Indija, Japonska, Kambodža, Pakistan …)
☐ Rom/-inja
☐ Drugo, kaj ……………………………………………………
☐ Ne vem

B) Technoference (Stockdale, Coyne, Padilla-Walker, 2018)

9. Kako pogosto si v zadnjem mesecu uporabljal/-a pametni telefon, ko si se hkrati pogovarjal/-a s prijateljem/-ico?
   - Nikoli
   - Redko
   - Včasih
   - Pogosto
   - Vedno

10. Spodaj je naštetih nekaj trditev o rabi mobilnih telefonov med pogovorom s prijatelji. Pri vsaki trditvi prosimo oceni, v kolikšni meri omenjeno vedenje velja zate. Ko ocenjuješ spodnje trditve imej v mislih zadnji mesec.

	Sploh ne drži	Ne drži	Deloma ne drži deloma drži	Drži	Popolnoma drži
Svoje prijatelje ignoriram, kadar uporabljam mobilni telefon.	☐	☐	☐	☐	☐

Moji prijatelji težko pridobijo mojo pozornost, kadar uporabljam mobilni telefon.	☐	☐	☐	☐	☐
Na svoj mobilni telefon pogledam tudi, če sem ravno sredi pogovora s svojimi prijatelji.	☐	☐	☐	☐	☐

C) Brief Measure of Social Media Self Control Failure (Du et al., 2018)

11. Kako pogosto si se v zadnjem mesecu vdal/-a želji po uporabi družbenih omrežij, čeprav ...

	Nikoli	Redko (1–3 krat)	Včasih (4–6 krat)	Pogosto (7–9 krat)	Zelo pogosto (10 krat ali več)	Zame ni relevant no
... je bilo to v takrat v nasprotju z drugimi cilji (npr. opravljanjem šolskih ali drugih nalog)?	☐	☐	☐	☐	☐	☐
... si takrat uporabljal/-a svoj čas manj učinkovito?	☐	☐	☐	☐	☐	☐
... si takrat odlašal/-a z drugimi stvarmi, ki si jih želel/-a ali moral/-a narediti?	☐	☐	☐	☐	☐	☐

D) Social media-induced inspiration scale (Meier & Schäfer, 2018)

12. Naslednje trditve se nanašajo na to, kako si se v zadnjem mesecu počutil/-a, ko si uporabljal/-a družbena omrežja. Pri vsaki trditvi prosimo oceni, v kolikšni meri se strinjaš oz. ne strinjaš, da opisano vedenje velja zate. Če ne uporabljaš družbenih omrežij, izberi možnost »Zame ni relevantno«.

	Sploh se ne strinjam	Se ne strinjam	Deloma se ne strinjam, deloma se strinjam	Se strinjam	Popolno ma se strinjam	Zame ni relevant no
Ko uporabljam družbena omrežja, se počutim navdihnjeno.	☐	☐	☐	☐	☐	☐
Ko uporabljam družbena omrežja, me objave drugih navdihujejo, da naredim nekaj [novega].	☐	☐	☐	☐	☐	☐

E) Posting positive social media content (short form) (Schreurs & Vandenbosch 2021)

13. Naslednje trditve se nanašajo na to, kako si se predstavljal/-a na družbenih omrežjih v zadnjem mesecu. Pri vsaki trditvi prosimo oceni, kako pogosto opisano vedenje velja zate. Če ne uporabljaš družbenih omrežij, izberi možnost »Zame ni relevantno«.

Kako pogosto na večinoma javnih aplikacijah, kot so družbena omrežja, objavljaš objave, na katerih ...

	Nikoli	Redko	Včasih	Pogosto	Zelo pogosto	Zame ni relevant no
... si videti lepo? (F2)	☐	☐	☐	☐	☐	☐
... pokažeš, da se zelo zabavaš? (F1)	☐	☐	☐	☐	☐	☐
... si videti uspešen/-na (npr. nekaj dosežeš v šoli ali pri hobiju)? (F3)	☐	☐	☐	☐	☐	☐
... pokažeš, kako odlično prijateljstvo imaš (npr. da je jasno videti, da se skupaj zelo zabavata)? (F1)	☐	☐	☐	☐	☐	☐
... počneš prijetne stvari (npr. si v kinu ali živalskem vrtu, na pijači/večerji s prijateljem/-ico)? (F1)	☐	☐	☐	☐	☐	☐
... pokažeš lep stil oblačenja? (F2)	☐	☐	☐	☐	☐	☐
... pokažeš, da si vesel/-a? (F1)	☐	☐	☐	☐	☐	☐
... pokažeš, da si na lepih počitnicah? (F1)	☐	☐	☐	☐	☐	☐

F) Internet aggression (Werner et al.2010)

14. Naslednje trditve se nanašajo na tvoje vedenje na Internetu (npr. na družbenih omrežjih). Pri vsaki trditvi prosimo oceni, kako pogosto je v zadnjih mesecu opisano vedenje veljalo zate na lestvici od »nikoli« do »5-krat ali več«. Če ne uporabljaš Interneta, izberi možnost »Zame ni relevantno«.

	Nikoli	1–2 krat	3–4 krat	5-krat ali več	Zame ni relevantno
Na Internetu sem nekomu grozil/-a ali ga/jo osramotil/-a (npr. z objavljanjem ali pošiljanjem sporočil o njej/njem tako, da so jih videli drugi).	☐	☐	☐	☐	☐
Drugim sem rekel, naj blokirajo sporočila od nekoga, ki mi ni bil/-a všeč ali sem bil/-a nanj/-o jezen/-na.	☐	☐	☐	☐	☐
Na Internetu sem se norčeval/-a iz nekoga ali sem jezil/-a nekoga, na katerega sem bil/-a jezen/-na.	☐	☐	☐	☐	☐
Na spletu sem nesramno ali grdo komentiral/-a o nekom drugem.	☐	☐	☐	☐	☐

G) The satisfaction of basic psychological needs in adolescents (Girelli et al 2019)

Naslednja vprašanja se nanašajo na tvoje življenje in tvoje občutke.

15. Pozorno preberi naslednje trditve in razmisli o svojem življenju. Prosimo, oceni, v kolikšni meri omenjene trditve veljajo zate.

	Sploh ne drži	Ne drži	Deloma ne drži deloma drži	Drži	Popolnoma drži
Menim, da se lahko sam/-a svobodno odločam o tem, kako naredim svoje stvari.	☐	☐	☐	☐	☐
Veliko stvari mi gre dobro od rok.	☐	☐	☐	☐	☐
Všeč so mi ljudje, s katerimi komuniciram.	☐	☐	☐	☐	☐
Menim, da se lahko sam/-a odločam, kako bom živel/-a svoje življenje.	☐	☐	☐	☐	☐
Lahko se naučim novih zanimivih veščin.	☐	☐	☐	☐	☐
Dobro se razumem z ljudmi, ki jih srečam.	☐	☐	☐	☐	☐

Na splošno mislim, da lahko svobodno izrazim, kaj zares mislim.	☐	☐	☐	☐	☐
Imam veliko priložnosti, da lahko pokažem kako sposoben/-na sem.	☐	☐	☐	☐	☐
Ljudi, s katerimi redno komuniciram, imam za svoje prijatelje.	☐	☐	☐	☐	☐
Zdi se mi, da sem bolj ali manj lahko to, kar sem.	☐	☐	☐	☐	☐
Ljudje mi pravijo, da sem dober/-ra v tem, kar počnem.	☐	☐	☐	☐	☐
Ljudje so na splošno precej prijazni do mene.	☐	☐	☐	☐	☐
