# Supplementary material for: Positive digital communication among youth: The development and validation of the digital flourishing scale for adolescents
Source: Front Digit Health. 2022 Sep 1;4:975557. doi: 10.3389/fdgth.2022.975557 (PMC9474732; doi:10.3389/fdgth.2022.975557)
Supplement: Supplementary file 3 [file Table_3.pdf]

Appendix C:

Table C: Study 1 number of respondents, communalities, and rotated factor loadings, reliability and descriptive statistics of the initial items of the DFSA Study 1

			Factor 1	Factor 2	Factor 3	Factor 4	Factor 5
	N	Communalities	Authentic self-presentation	Positive social comparison	Civil participation	Connectedness	Self-control
I show my true self online.	131	.524	.765				
When communicating online, I feel comfortable presenting the person I am.	131	.599	.742				
What I post online reflects who I really am.	126	.493	.762				
I feel comfortable presenting who I truly am online, in the same way I do offline.	133	.665	.793				
I allow people who I connect with online to see who I really am.	130	.533	.773				
Seeing others' achievements online inspires me to do better.	138	.500		.813			
Seeing how others present themselves online motivates me to make changes in my own life.	137	.684		.851			
Comparing myself to others online motivates me to accomplish the things I want in life.	138	.654		.749			
I compare my life to those people online (e.g., peers, influencers) who are going to push me to be better.	134	.516		.681			
When I talk to others online, I know how to share my point of view without offending them.	134	.332			.627		
When I communicate online, I am careful to adapt my comments and behaviors to be appropriate for whoever will read them (e.g., my friends, my teacher, my parents, younger children).	138	.530			.877		
When I talk to others online about something important to me, I know how to stand for it in a polite manner.	135	.378			.608		
When I talk to others online about politics (e.g., about the government, the President, elections), I know how to do it politely.	100	.604			.779		
When something that others say or do online makes me feel angry, I am able to respond in a calm way.	129	.482			.671		
I feel part of a group when I communicate with others online.	139	.681				.785	

I find my online communication (e.g., chatting with peers, playing online games with others) very important.	141	.515				.756	
I feel closely connected to the groups I connect with online.	139	.575				.821	
I communicate online when I want to, not when notifications tell me to.*	138	.038				.579	.449
I feel in control of when to start and when to stop spending time on online communication.	134	.409					.602
For the most part, I feel in control of how much time I spend communicating with others online (e.g., chatting with friends, posting on Instagram, playing online games with others).	135	.414					.707
When I browse through online content, I feel in control of how I spend my time.	138	.278					.605
I am able to disconnect from my online communication when I need a break.	136	.422					.676
When I browse (scroll) through my online apps (like Snapchat, TikTok, Instagram etc.), I feel connected to others.**	139	.227					
I could turn to people who I connect with online (e.g., acquaintances), if I needed advice on a problem.**	136	.327					
I compare my life to those people online (e.g., peers, influencers) who I can learn from.**	141	.320					
Eigenvalues			6.599	3.395	2.854	2.052	1.947
% of variance			13.5	11.4	11.1	10.9	9.4
M (SD)			3.4 (.87)	3.2 (.83)	4 (.70)	3.2 (.75)	3.7 (.67)
Cronbach's α			.86	.84	.81	.76	.72

Note. Loadings <.500 are not shown.
* Item was removed because of loading on the theoretically incorrect factor.
** Item was removed due to low factor loadings.
