# Supplementary material for: Positive digital communication among youth: The development and validation of the digital flourishing scale for adolescents
Source: Front Digit Health. 2022 Sep 1;4:975557. doi: 10.3389/fdgth.2022.975557 (PMC9474732; doi:10.3389/fdgth.2022.975557)
Supplement: Supplementary file 4 [file Table_4.pdf]

Appendix D:

Table D: Correlation matrix between the factors of DFSA (Study 1)

	Connectedness	Civil participation	Positive social comparison	Authentic self-presentation	Self-control
Connectedness	1	.14	.54***	.23*	.017
Civil participation		1	.29**	.09	.19
Positive social comparison			1	.37***	.068
Authentic self-presentation				1	.21*
Self-control					1

Note. ***p < .001; **p < .01; *p < .05
