# Supplementary material for: Positive digital communication among youth: The development and validation of the digital flourishing scale for adolescents
Source: Front Digit Health. 2022 Sep 1;4:975557. doi: 10.3389/fdgth.2022.975557 (PMC9474732; doi:10.3389/fdgth.2022.975557)
Supplement: Supplementary file 5 [file Table_5.pdf]

DFSA scale and scales to measure construct validity Study 2

Appendix E: Final DFSA Scale in English (Study 2)

The following questions are all concerned with learning more about your online communication habits and experiences during the past four months. We refer to online communication to include conversations and participation across a variety of online applications. Examples of online communication are emailing, chatting (e.g., smartphone SMS texting, Direct messaging tools like WhatsApp, Viber), interacting on social media (e.g., posting, commenting or liking posts on SnapChat, Instagram, TikTok or YouTube), phone conversations, FaceTime, zoom etc.

There is no right or wrong answer. We are just interested in your most truthful opinions.

Please indicate how true the following behaviors were for you in the past four months using the options from "Not at all true of me" to "Very true of me". If you never engaged in one of the online communication behaviors listed below, please select "Not applicable to me".

The following statements are about your experiences with connecting with others online. When assessing the statements think about the past four months.

	Not at all true of me	Not true of me	Partly not true, partly true of me	True of me	Very true of me	I don't know/ Not applicable to me
I feel part of a group when I communicate with others online.	☐	☐	☐	☐	☐	☐
I find my online communication (e.g., chatting with peers, playing online games with others) very important.	☐	☐	☐	☐	☐	☐
I feel closely connected to the groups I connect with online.	☐	☐	☐	☐	☐	☐

The following statements are about how you express your opinion online. When assessing the statements think about the past four months.

	Not at all true of me	Not true of me	Partly not true, partly true of me	True of me	Very true of me	I don't know/ Not applicable to me
When I talk to others online, I know how to share my point of view without offending them.	☐	☐	☐	☐	☐	☐

When I communicate online, I am careful to adapt my comments and behaviors to be appropriate for whoever will read them (e.g., my friends, my teacher, my parents, younger children).	☐	☐	☐	☐	☐	☐
When I talk to others online about something important to me, I know how to stand up for it in a polite manner.	☐	☐	☐	☐	☐	☐
When I talk to others online about politics (e.g., about the government, the President, elections), I know how to do it politely.	☐	☐	☐	☐	☐	☐
When something that others say or do online makes me feel angry, I am able to respond in a calm way.	☐	☐	☐	☐	☐	☐

The following statements are about comparing yourself to others on social media or other online applications. When assessing the statements think about the past four months.

	Not at all true of me	Not true of me	Partly not true, partly true of me	True of me	Very true of me	I don’t know/ Not applicable to me
Seeing others’ achievements online inspires me to do better.	☐	☐	☐	☐	☐	☐
Seeing how others present themselves online motivates me to make changes in my own life.	☐	☐	☐	☐	☐	☐
Comparing myself to others online motivates me to accomplish the things I want in life.	☐	☐	☐	☐	☐	☐
I compare my life to those people online (e.g., peers, influencers) who are going to push me to be better.	☐	☐	☐	☐	☐	☐

The following statements are about how you present yourself online. When assessing the statements think about the past four months.

	Not at all true of me	Not true of me	Partly not true, partly true of me	True of me	Very true of me	I don’t know/ Not applicable to me

I show my true self online.	☐	☐	☐	☐	☐	☐
When communicating online, I feel comfortable presenting the person I am.	☐	☐	☐	☐	☐	☐
What I post online reflects who I really am.	☐	☐	☐	☐	☐	☐
I feel comfortable presenting who I truly am online, in the same way I do offline.	☐	☐	☐	☐	☐	☐
I allow people who I connect with online to see who I really am.	☐	☐	☐	☐	☐	☐

The following statements are about the control you have to have about online communication. When assessing the statements think about the past four months.

	Not at all true of me	Not true of me	Partly not true, partly true of me	True of me	Very true of me	I don’t know/ Not applicable to me
I feel in control of when to start and when to stop spending time on online communication.	☐	☐	☐	☐	☐	☐
For the most part, I feel in control of how much time I spend communicating with others online (e.g., chatting with friends, posting on Instagram, playing online games with others).	☐	☐	☐	☐	☐	☐
When I browse through online content, I feel in control of how I spend my time.	☐	☐	☐	☐	☐	☐
I am able to disconnect from my online communication when I need a break.	☐	☐	☐	☐	☐	☐

Appendix E: Final DFSA Scale in Slovenian (Study 2)

Z naslednjimi vprašanji želimo izvedeti več o tvojih navadah in izkušnjah s komuniciranjem preko spleta v zadnjih štirih mesecih.

Z izrazom »komuniciranje preko spleta« mislimo na pogovore in udeležbo na različnih spletnih aplikacijah. Na primer na e-pošto, klepetanje (npr. s SMS sporočili, z neposrednimi sporočili npr. preko Whatsappa), komunikacijo na družbenih omrežjih (npr. objavljanje, komentiranje ali všečkanje objav na SnapChatu, Instagramu, TikToku, Youtube-u), pa tudi telefonske pogovore, FaceTime, Zoom in drugo.

Ni pravilnih ali napačnih odgovorov, zanima nas le tvoje iskreno mnenje.

Prosimo, oceni, v kolikšni meri je v zadnjih štirih mesecih vsaka trditev držala zate, tako da izbereš eno izmed možnosti od zame »Sploh ne drži« do »Popolnoma drži«. Če katerega od omenjenih vedenj na spletu nisi počel/-a, izberi možnost »Se ne nanaša«.

Naslednje trditve se nanašajo na tvoje izkušnje glede povezovanja z drugimi na spletu. Ko ocenjuješ spodnje trditve, imej v mislih zadnje štiri mesece.

	Sploh ne drži	Ne drži	Deloma ne drži deloma drži	Drži	Popolnoma drži	Ne vem/ Se ne nanaša
Ko komuniciram z drugimi preko spleta, se počutim kot del skupine na spletu.	☐	☐	☐	☐	☐	☐
Zdi se mi, da je moje komuniciranje preko spleta (npr. pogovori s prijatelji, igranje iger z drugimi preko spleta) zame zelo pomembno.	☐	☐	☐	☐	☐	☐
Počutim se tesno povezanega/-o s skupinami, s katerimi se družim na spletu.	☐	☐	☐	☐	☐	☐

Naslednje trditve se nanašajo na to, kako si izražal/-a svoje mnenje na spletu. Ko ocenjuješ spodnje trditve, imej v mislih zadnje štiri mesece.

	Sploh ne drži	Ne drži	Deloma ne drži deloma drži	Drži	Popolnoma drži	Ne vem/ Se ne nanaša
Ko se pogovarjam z drugimi preko spleta, znam izraziti mnenje, brez da sem pri tem žaljiv/-a.	☐	☐	☐	☐	☐	☐

Kadar komuniciram preko spleta, pazim, da prilagodim svoje komentarje in vedenje na način, da so primerni za vse, ki bi jih prebrali (npr. moji prijatelji, učitelji, starši, mlajši otroci).	☐	☐	☐	☐	☐	☐
Ko se pogovarjam z drugimi preko spleta o nečem, kar mi veliko pomeni, znam na spoštljiv način to tudi zagovarjati.	☐	☐	☐	☐	☐	☐
Ko se pogovarjam z drugimi preko spleta o politiki (npr. o vladi, predsedniku, volitvah, …) znam pri tem biti spoštljiv/-a.	☐	☐	☐	☐	☐	☐
Kadar drugi na spletu rečejo ali storijo nekaj, kar me razjezi, sem se na to zmožen/-na odzvati na miren način.	☐	☐	☐	☐	☐	☐

Naslednje trditve se nanašajo na to, kako si se primerjal/-a z drugimi na družbenih omrežjih in drugih spletnih aplikacijah. Ko ocenjuješ spodnje trditve, imej v mislih zadnje štiri mesece.

	Sploh ne drži	Ne drži	Deloma ne drži deloma drži	Drži	Popolno ma drži	Ne vem/ Se ne nanaša
Dosežki drugih, ki jih vidim na spletu, me navdihnejo, da postanem boljši/-a.	☐	☐	☐	☐	☐	☐
To, da vidim, kako se drugi predstavljajo na spletu, me motivira, da sprejmem spremembe v svojem življenju.	☐	☐	☐	☐	☐	☐
To, da se primerjam z drugimi na spletu, me motivira, da dosežem stvari, ki jih želim v življenju.	☐	☐	☐	☐	☐	☐

Na spletu primerjam svoje življenje s tistimi ljudmi (npr. sovrstniki, vplivneži), ki me spodbudijo, da sem boljši/-a.	☐	☐	☐	☐	☐	☐

Naslednje trditve se nanašajo na to, kako si se predstavljal/-a drugim na spletu. Ko ocenjuješ spodnje trditve, imej v mislih zadnje štiri mesece.

	Sploh ne drži	Ne drži	Deloma ne drži deloma drži	Drži	Popolno ma drži	Ne vem/ Se ne nanaša
Na spletu pokažem pravega/-o sebe.	☐	☐	☐	☐	☐	☐
Ko komuniciram preko spleta, se brez zadržkov predstavim takega/-o kot sem.	☐	☐	☐	☐	☐	☐
Kar objavim na spletu, odraža to, kdo jaz v resnici sem.	☐	☐	☐	☐	☐	☐
Na spletu brez zadržkov pokažem pravega/-o sebe, na enak način kot to storim v resničnem življenju.	☐	☐	☐	☐	☐	☐
Ljudem, s katerimi se družim preko spleta, dovolim, da me vidijo takega/-o kot resnično sem.	☐	☐	☐	☐	☐	☐

Naslednje trditve se nanašajo na nadzor tvojega komuniciranja na spletu. Ko ocenjuješ spodnje trditve, imej v mislih zadnje štiri mesece.

	Sploh ne drži	Ne drži	Deloma ne drži deloma drži	Drži	Popolno ma drži	Ne vem/ Se ne nanaša
Zdi se mi, da lahko nadzorujem, kdaj začnem in kdaj preneham s komuniciranjem preko spleta.	☐	☐	☐	☐	☐	☐

Zdi se mi, da večinoma lahko nadzorujem, koliko časa porabim za komuniciranje preko spleta z drugimi (npr. klepetanje s prijatelji, objavljanje prispevkov na Instagramu, igranje igric z drugimi preko spleta).	☐	☐	☐	☐	☐	☐
Ko brskam po vsebinah na spletu, se mi zdi, da imam nadzor nad tem, kako preživljam svoj čas.	☐	☐	☐	☐	☐	☐
Zmožen/-na sem se odklopiti od svojega komuniciranja preko spleta, kadar potrebujem odmor.	☐	☐	☐	☐	☐	☐

Appendix E: Scales used to estimate construct validity in English (Study 2)

A) **DEMOGRAPHIC VARIABLES**

PART 1: Basic questions about you and your family.

1. What is your name and surname?

Note that this information together with your birthdate is stored separately from your answers in this survey to ensure your anonymity.

2. In which year were you **born**?

(dropdown 2000-2011)

3. I am a:

☐ Boy
☐ Girl
☐ Other
☐ Prefer not to say

4. What is the name of your **school**?

☐ II. osnovna šola Celje (1)
☐ Osnovna šola Antona Martina Slomška Vrhnika (2)
☐ Osnovna šola Brežice (3)
☐ Osnovna šola Bršljin Novo mesto (4)
☐ Osnovna šola Danile Kumar Ljubljana Bežigrad (5)
☐ Osnovna šola Dragomirja Benčiča - Brkina Hrpelje (6)
☐ Osnovna šola Ivana Cankarja Vrhnika (7)
☐ Osnovna šola Kidričevo (8)

☐ Osnovna šola Milojke Štrukelj Nova Gorica (9)
☐ Osnovna šola Ob Rinži Kočevje (10)
☐ Osnovna šola Pohorskega odreda Slovenska Bistrica (11)
☐ Osnovna šola Puconci (12)
☐ Osnovna šola Šentjanž pri Dravogradu (13)
☐ Osnovna šola Simona Gregorčiča Kobarid (14)
☐ Osnovna šola Stopiče (15)
☐ Osnovna šola Tončke Čeč Trbovlje (16)
☐ Osnovna šola Toneta Čufarja Jesenice (17)
☐ Ekonomska gimnazija in srednja šola Radovljica (18)
☐ Ekonomska šola Ljubljana (19)
☐ Gimnazija Jožeta Plečnika Ljubljana (20)
☐ Gimnazija Kranj (21)
☐ Šolski center Celje. Srednja šola za storitvene dejavnosti in logistiko (22)
☐ Šolski center Nova Gorica. Strojna, prometna in lesarska šola Nova Gorica (23)
☐ Srednja ekonomska šola in gimnazija Maribor (24)
☐ Srednja gozdarska in lesarska šola Postojna (25)
☐ Srednja šola za oblikovanje in fotografijo Ljubljana (26)
☐ Srednja tehniška šola Koper (27)
☐ Other (specify): (99) ____

5. What **class** are you in?: (choice) ☐6 ☐7 ☐8 ☐9 ☐1 ☐2 ☐3 ☐4 other: ____
6. Which **educational tract** do you follow?

☐ Secondary vocational education (e.g. carpenter, merchant, metalworker – toolmaker)
☐ Secondary professional-technical education (e.g. economic technician, mechanical technician, pre-school teacher)
☐ Secondary general education (e.g. general high school, economic high school)

7. What is your **ethnic** background? *You can choose multiple options*

☐ Central European (e.g. Germany, Austria, Slovenia, Poland, Hungary)
☐ West European (e.g. Belgium, France, United Kingdom, The Netherlands)
☐ East European (e.g. Russia, Ukraine, Belarus)
☐ South European (e.g. Italy, Spain, Portugal)
☐ South-East European (e.g. Croatia, Serbia, North Macedonia, Bulgaria, Romania)
☐ North European (e.g. Sweden, Finland, Norway)
☐ African or Middle-East (e.g. Morocco, Egypt, Congo…)
☐ North-American (Canada or USA)
☐ South-American or Latin American (e.g. Cuba, Mexico, Brazil…)
☐ Asian (e.g. China, India, Japan, Cambodia, Pakistan, …)
☐ Romani
☐ Other, specify: …………………………………………………
☐ I don't know

8. What is the highest degree that **your dad (or male guardian)** obtained?

☐ Unfinished primary education
☐ Primary Education
☐ Secondary Education
☐ Higher vocational education
☐ Professional higher education and University (Bachelor, Master's, PhD)
☐ I don't know, but my dad works as a:
☐ I can't respond

9. What is the highest degree that **your mom (or female guardian)** obtained?

☐ Unfinished primary education
☐ Primary Education
☐ Secondary Education
☐ Higher vocational education
☐ Professional higher education and University (Bachelor, Master's, PhD)
☐ I don't know, but my mom works as a:
☐ I can't respond

B) ATTENTION CHECK

10. We would like to know if you are still filling in the survey attentively. If you are still attentive in answering the questionnaire, please answer "Yes, I am still attentive to answer the questionnaire".

☐ Yes, I am still attentive to answer the questionnaire.
☐ No, I am not attentive and I answer the questions randomly

C) The Secure Attachment Style subscale of the Short form of the Adolescent Friendship Attachment Scale (S)

11. Think of someone you feel closest to above all others. This person should be close to your own age. They might be your best friend or someone from one of your classes, sports teams, or even just someone you hang around with sometimes. Indicate how much you agree or disagree with the following statements.

		Strongly disagree	Disagree	Partly disagree partly agree	Agree	Strongly agree
**I think it would be difficult to replace my friend.**	**S**					
**I can trust my friend.**	**S**					
**I know that my friend is loyal.**	**S**					
**I enjoy spending time with my friend.**	**S**					
**I know I can rely on my friend.**	**S**					
I worry my friend doesn't really like me.	An					

I am not sure I can always depend on my friend.	An					
I would like my friend to be more understanding.	An					
I worry about becoming too close to my friend.	An					
I don't feel as close to my friend as I would like.	An					
I let my friend know about things that trouble me.	Av r					
I don't turn to my friend for support when things are difficult.	Av					
I seek out my friend when things go wrong.	Av r					
I don't need to rely on my friend.	Av					
Without this friendship, it would be very hard to cope when things are difficult.	Av r					

D) Authenticity of Posted Positive Content

The following questions are about what you post and what you see on social media.

Interactions with Positive Social Media Content Scales

12. The next questions are about **posts and stories on social media platforms** that you shared with many people in the past 4 months (think about for instance, followers on Instagram, Snapchat, or TikTok, but not messages on WhatsApp). These posts and stories can be seen by relatively many people, typically you don't know all of these people equally well.

In the past 4 months, when you posted such posts or stories, how often were they messages on which you...

	Never	Rarely	Sometimes	Often	Very often
… looked **beautiful**	☐	☐	☐	☐	☐
… showed that you had a lot of **fun**	☐	☐	☐	☐	☐
… looked **successful** (e.g., you had achieved something at school or in a hobby)	☐	☐	☐	☐	☐

… showed how great a **friendship** was (e.g., that it was clear you had much fun together)	☐	☐	☐	☐	☐
… did **nice things** (e.g., went to the movie theaters or to the zoo, had something to drink/eat with a friend)	☐	☐	☐	☐	☐
… showed a **nice clothing style**	☐	☐	☐	☐	☐
… showed that you were **happy**	☐	☐	☐	☐	☐
… showed a **nice holiday** you had done	☐	☐	☐	☐	☐

<u>Authenticity of posted positive content</u>

13. When you posted the posts or stories described in the previous question on social media applications how often did you have the impression that …

	Never	Rarely	Sometimes	Often	Very often
... these posts showed who you really are.	☐	☐	☐	☐	☐

Appendix E: Scales used to estimate construct validity in Slovenian (Study 2)

A) DEMOGRAPHIC VARIABLES

PRVI DEL: Osnovna vprašanja o tebi in tvoji družini.

1. Vnesi svoje ime in priimek.

**Tvoje ime in priimek bomo skupaj z letnico rojstva shranili ločeno od tvojih odgovorov in bomo zagotovili anonimnost podatkov.*

2. Kdaj si rojen/-a? Leta …….

➔ (dropdown 2000-2011)

3. Sem:

☐ Fant
☐ Dekle
☐ Drugo
☐ Ne želim odgovoriti

4. Katero **šolo** obiskuješ?

☐ Boy
☐ Girl
☐ Other
☐ Prefer not to say

5. What is the name of your **school**?

☐ II. osnovna šola Celje (1)
☐ Osnovna šola Antona Martina Slomška Vrhnika (2)
☐ Osnovna šola Brežice (3)
☐ Osnovna šola Bršljin Novo mesto (4)
☐ Osnovna šola Danile Kumar Ljubljana Bežigrad (5)
☐ Osnovna šola Dragomirja Benčiča - Brkina Hrpelje (6)
☐ Osnovna šola Ivana Cankarja Vrhnika (7)
☐ Osnovna šola Kidričevo (8)
☐ Osnovna šola Milojke Štrukelj Nova Gorica (9)
☐ Osnovna šola Ob Rinži Kočevje (10)
☐ Osnovna šola Pohorskega odreda Slovenska Bistrica (11)
☐ Osnovna šola Puconci (12)
☐ Osnovna šola Šentjanž pri Dravogradu (13)
☐ Osnovna šola Simona Gregorčiča Kobarid (14)
☐ Osnovna šola Stopiče (15)
☐ Osnovna šola Tončke Čeč Trbovlje (16)
☐ Osnovna šola Toneta Čufarja Jesenice (17)
☐ Ekonomska gimnazija in srednja šola Radovljica (18)
☐ Ekonomska šola Ljubljana (19)
☐ Gimnazija Jožeta Plečnika Ljubljana (20)

☐ Gimnazija Kranj (21)
☐ Šolski center Celje. Srednja šola za storitvene dejavnosti in logistiko (22)
☐ Šolski center Nova Gorica. Strojna, prometna in lesarska šola Nova Gorica (23)
☐ Srednja ekonomska šola in gimnazija Maribor (24)
☐ Srednja gozdarska in lesarska šola Postojna (25)
☐ Srednja šola za oblikovanje in fotografijo Ljubljana (26)
☐ Srednja tehniška šola Koper (27)
☐ Drugo (vnesi): (99) ____

6. V katerem **razredu** / **letniku** si?: ☐6 ☐7 ☐8 ☐9 ☐1 ☐2 ☐3 ☐4 Drugo (vnesi):

7. V kateri **program izobraževanja** si vpisan/-a?

☐ Srednje poklicno izobraževanje (npr. mizar, gozdar, frizer, oblikovalec kovin – orodjar, mehatronik operater, bolničar negovalec)
☐ Srednje strokovno-tehniško izobraževanje (npr. ekonomski tehnik, strojni tehnik, medijski tehnik, gozdarski tehnik, logistični tehnik, ustvarjalec modnih oblačil)
☐ Srednje splošno izobraževanje (npr. gimnazija, ekonomska gimnazija)

8. Kako bi opisal svojo **etnično pripadnost**? Izbereš lahko več odgovorov.

☐ Srednji/-a Evropejec/-ka (npr. Nemčija, Avstrija, Slovenija, Poljska, Madžarska …)
☐ Zahodni/-a Evropejec/-ka (npr. Belgija, Francija, Velika Britanija, Nizozemska …)
☐ Vzhodni/-a Evropejec/-ka (npr. Rusija, Ukrajina, Belorusija …)
☐ Južni/-a Evropejec/-ka (npr. Italija, Španija, Portugalska …)
☐ Jugovzhodni/-a Evropejec/-ka (npr. Hrvaška, Srbija, Severna Makedonija, Bolgarija, Romunija …)
☐ Severni/-a Evropejec/-ka (npr. Švedska, Norveška, Finska …)
☐ Afričan/-ka ali prebivalec/-ka Bližnjega vzhoda (npr. Maroko, Egipt, Kongo …)
☐ Severnoameričan/-ka (Kanada ali Združene države Amerike)
☐ Južnoameričan/-ka ali prebivalec/-ka Latinske Amerike (npr. Kuba, Mehika, Brazilija …)
☐ Azijec/-ka (npr. Kitajska, Indija, Japonska, Kambodža, Pakistan …)
☐ Rom/-inja
☐ Drugo, kaj ……………………………………………………
☐ Ne vem

9. Katera je najvišja dosežena izobrazba tvojega **očeta** (oz. skrbnika)?

☐ Nedokončana osnovnošolska izobrazba
☐ Osnovnošolska izobrazba
☐ Srednješolska izobrazba
☐ Višješolska izobrazba
☐ Visokošolska in univerzitetna izobrazba (diploma, magisterij, doktorat)
☐ Ne vem, ampak moj oče je zaposlen kot _____
☐ Ne morem odgovoriti

10. Katera je najvišja dosežena izobrazba tvoje **mame** (oz. skrbnice)?

☐ Nedokončana osnovnošolska izobrazba
☐ Osnovnošolska izobrazba

☐ Srednješolska izobrazba
☐ Višješolska izobrazba
☐ Visokošolska in univerzitetna izobrazba (diploma, magisterij, doktorat)
☐ Ne vem, ampak moja mama je zaposlena kot _____
☐ Ne morem odgovoriti

B) The Secure Attachment Style subscale of the Short form of the Adolescent Friendship Attachment Scale (S)

1. Pomisli na osebo, ki ti je najbližje v primerjavi s katerokoli drugo osebo. Ta oseba bi morala biti približno tvojih let. Morda je to tvoj/-a najboljši/-a prijatelj/-ica, sošolec/-ka, nekdo iz iste športne ekipe ali celo nekdo, s katerim se družiš le občasno. Navedi, v kolikšni meri se strinjaš ali ne strinjaš z naslednjimi trditvami.

		Sploh se ne strinjam	Se ne strinjam	Deloma se ne strinjam, deloma se strinjam	Se strinjam	Popolnoma se strinjam
**Zdi se mi, da bi bilo prijatelja/-ico težko nadomestiti.**	**S**					
**Prijatelju/-ici lahko zaupam.**	**S**					
**Vem, da mi je prijatelj/-ica zvest/-a.**	**S**					
**Pri preživljanju prostega časa s prijateljem/-ico uživam.**	**S**					
**Vem, da se na prijatelja/-ico lahko zanesem.**	**S**					
Skrbi me, da me moj/-a prijatelj/-ica v resnici ne mara.	An					
Nisem prepričan/-a, da se vedno lahko zanesem na svojega/-o prijatelja/-ico.	An					
Želim si, da bi bil/-a moj/-a prijatelj/-ica bolj razumevajoč/-a.	An					
Skrbi me, da bi se preveč navezal/-a na svojega/-o prijatelja/-ico.	An					
Svojemu/-i prijatelju/-ici se ne čutim tako blizu, kot bi želel/-a.	An					

Svojemu/-i prijatelju/-ici zaupam svoje težave.	Av					
Ko mi je težko, se ne obrnem k svojemu/-i prijatelju/-ici po podporo.	Av r					
Ko sem v težavah, iščem podporo pri svojem/-i prijatelju/-ici.	Av r					
Ni se mi treba zanašati na svojega/-o prijatelja/-ico.	Av					
Brez tega prijateljstva bi se mi bilo težko spoprijeti s težavami.	Av r					

C) Authenticity of Posted Positive Content

<u>Naslednja vprašanja se nanašajo na to, kar objavljaš in vidiš na družbenih omrežjih.</u>

Interactions with Positive Social Media Content Scales

1. Naslednja vprašanja se nanašajo na **objave in zgodbe na družbenih omrežjih**, ki si jih v zadnjih štirih mesecih **delil/-a z veliko ljudm**i. Običajno vseh teh ljudi ne poznaš enako dobro. (Pomisli na primer na sledilce na Instagramu, Snapchatu ali TikToku, ne pa sporočila na WhatsAppu).

Pri vsaki trditvi prosimo oceni, ko si <u>v zadnjih štirih mesecih</u> objavljal/-a objave ali storije, **kako pogosto so bile te objave sporočila, na katerih ...**

	Nikoli	Redko	Včasih	Pogosto	Zelo pogosto
... si videti **lepo**?	☐	☐	☐	☐	☐
... pokažeš, da se zelo **zabavaš**?	☐	☐	☐	☐	☐
... si videti **uspešen/-na** (npr. nekaj dosežeš v šoli ali pri hobiju)?	☐	☐	☐	☐	☐
... pokažeš, kako odlično **prijateljstvo** imaš (npr. da je jasno videti, da se skupaj zelo zabavata)?	☐	☐	☐	☐	☐
... počneš **prijetne stvari** (npr. si v kinu ali živalskem vrtu, na pijači/večerji s prijateljem/-ico)?	☐	☐	☐	☐	☐
... pokažeš lep **stil oblačenja**?	☐	☐	☐	☐	☐
... pokažeš, da si **vesel/-a**?	☐	☐	☐	☐	☐

... pokažeš, da si na **lepih počitnicah**?	☐	☐	☐	☐	☐

Authenticity of posted positive content

2. Pomisli na svoje **objave** in **zgodbe** na družbenih omrežjih**, opisane v prejšnjem vprašanju**. Prosimo oceni, v kako pogosto se ti zdi, da …

	Nikoli	Redko	Včasih	Pogosto	Zelo pogosto
... te objave pokažejo, kdo v resnici si.	O	O	O	O	O
