# Supplementary material for: Positive digital communication among youth: The development and validation of the digital flourishing scale for adolescents
Source: Front Digit Health. 2022 Sep 1;4:975557. doi: 10.3389/fdgth.2022.975557 (PMC9474732; doi:10.3389/fdgth.2022.975557)
Supplement: Supplementary file 6 [file Table_6.pdf]

Appendix F

Table F: Correlation matrix between the factors of DFSA (Study 2)

	Connectedness	Civil participation	Positive social comparison	Authentic self-presentation	Self-control
Connectedness	1	.094*	.30***	.24***	-.019
Civil participation		1	.26***	.32***	.38***
Positive social comparison			1	.22***	.096**
Authentic self-presentation				1	.21***
Self-control					1

Note. ***p < .001; **p < .01; *p < .05
